# Effects of *Lycium barbarum* Polysaccharides on Immunity and Metabolic Syndrome Associated with the Modulation of Gut Microbiota: A Review

**DOI:** 10.3390/foods11203177

**Published:** 2022-10-12

**Authors:** Cui Cao, Zhongfu Wang, Guiping Gong, Wenqi Huang, Linjuan Huang, Shuang Song, Beiwei Zhu

**Affiliations:** 1 Collaborative Innovation Center of Seafood Deep Processing, National Engineering Research Center of Seafood, School of Food Science and Technology, Dalian Polytechnic University, Dalian 116034, China; 2 National & Local Joint Engineering Laboratory for Marine Bioactive Polysaccharide Development and Application, Dalian Polytechnic University, Dalian 116034, China; 3 Shaanxi Natural Carbohydrate Resource Engineering Research Center, College of Food Science and Technology, Northwest University, Xi’an 710069, China

**Keywords:** *Lycium barbarum* polysaccharides, structural characteristics, gut microbiota, immunity, metabolic syndrome

## Abstract

*Lycium barbarum* polysaccharides (LBPs) have attracted increasing attention due to their multiple pharmacological activities and physiological functions. Recently, both in vitro and in vivo studies have demonstrated that the biological effects of dietary LBPs are related to the regulation of gut microbiota. Supplementation with LBPs could modulate the composition of microbial communities, and simultaneously influence the levels of active metabolites, thus exerting their beneficial effects on host health. Interestingly, LBPs with diverse chemical structures may enrich or reduce certain specific intestinal microbes. The present review summarizes the extraction, purification, and structural types of LBPs and the regulation effects of LBPs on the gut microbiome and their derived metabolites. Furthermore, the health promoting effects of LBPs on host bidirectional immunity (e.g., immune enhancement and immune inflammation suppression) and metabolic syndrome (e.g., obesity, type 2 diabetes, and nonalcoholic fatty liver disease) by targeting gut microbiota are also discussed based on their structural types. The contents presented in this review might help to better understand the health benefits of LBPs targeting gut microbiota and provide a scientific basis to further clarify the structure–function relationship of LBPs.

## 1. Introduction

The human gut microbiota is a complex and abundant community composed of up to 10^14^ microorganisms with about 1150 species [1]. The community is dominated by Firmicutes and Bacteroidetes, which account for more than 80–90%, and then followed by Proteobacteria, Actinobacteria, Verrucomicrobia, Fusobacteria, Cyanobacteria, and Spirochaetes as minor components [2]. The gut microbiota is regarded as a neglected human organ to some extent in the human–microbe superorganism [3]. Furthermore, the dysbiosis of gut microbiota not only affects the host physiological functions (e.g., nutrient digestion, absorption, and metabolism), but triggers diseases (e.g., immune dysregulation responses and metabolic syndrome) [4,5,6]. Therefore, the balance of gut microbiota, including microbial diversity, richness, composition, and functionality, is critical for the health of the host. Numerous studies have demonstrated that several factors, such as genetics, antibiotics, age, and diet, can influence the gut microbiome [6,7]. Among these factors, a short-term diet can lead to significant microbial changes. More importantly, non-digestible polysaccharides can be degraded and utilized by gut microbiota instead of the host, which encode the carbohydrate active enzymes (CAZymes), such as glycoside hydrolases (GHs), polysaccharide lyases (PLs), glycosyltransferases (GTs) and carbohydrate esterases (CEs), thereby improving beneficial metabolites (e.g., SCFAs) [8,9].

*Lycium barbarum*, also named Goji berry, Gouqizi, and wolfberry, is a perennial shrubbery of Solanaceae that is widely cultivated in China, Japan, Korea, North America, and Europe [10]. Currently, China is the largest supplier in the world, and a majority of *L. barbarum* fruits are distributed in the northwest regions of China, such as Ningxia, Xinjiang, Tibet, Inner Mongolia, Qinghai, and Gansu [11,12]. Notably, *L. barbarum* fruits from Ningxia region are the only species included in the Pharmacopoeia of the People’s Republic of China for many years due to their excellent quality [13]. Various bioactive constituents have been isolated and identified from *L. barbarum* fruits, including polysaccharides, carotenoids, vitamins, flavonoids, alkaloids, anthraquinones, anthocyanins, and organic acids. Among them, the polysaccharides, accounting for 5–8% of dried fruits, have been recognized one of the principal active components [10]. In recent decades, a great deal of research has now confirmed that *L. barbarum* polysaccharides (LBPs) have various biological functions, such as immunoregulation, anti-inflammation, anti-tumor activities, hypoglycemic/lipidemic activities, and retinal protection [14,15,16,17,18,19]. LBPs mainly include arabinogalactans, acidic heteropolysaccharides, glucans, and other polysaccharides [20,21,22,23,24]. Increasing evidence suggests that the molecular weight, monosaccharide composition, and glycosidic linkage of LBPs could influence their bioactivities, although the structure–activity relationship of polysaccharides is not yet clear. Therefore, elucidating the structures of LBPs would be beneficial to understand the mechanisms of their health effects and further develop their industrial application. However, many studies have shown that most LBPs are resistant to human digestive enzymes and can almost entirely reach the colon where they are digested and metabolized by gut microbiota, indicating that gut microbiota plays a crucial role in the beneficial effects of LBPs [25,26]. Currently, although the extraction, purification, structural characterization, and functional activities of LBPs have been summarized and reviewed [27,28,29], few reviews have discussed their structural types and summarized the modulation of LBPs on gut microbiota and the role of gut microbiota in the health effects of LBPs, as well as their potential mechanism based on their structural types.

This review mainly summarizes the modulation of LBPs on gut microbes and related metabolites. Furthermore, the protective effects of LBPs mediated by gut microbiota on immunoregulation (e.g., immunopotentiation and anti-inflammation), metabolic disease (e.g., obesity, type 2 diabetes, and nonalcoholic fatty liver disease), and other diseases (e.g., asthma and emotional impairment) have been summarized and discussed in order to better understand the health benefits of LBPs targeting gut microbiota in the present review. In addition, the current issues and future prospects for the relationship between the structure and function of LBPs are also discussed.

## 2. Isolation and Structure of LBPs

The elucidation of precise structures of LBPs is the prerequisite to unraveling the relationships between structures and functions. Numerous studies have demonstrated that the biological activities of LBPs are principally related to their primary and advanced structures [10,28]. Actually, the current studies mainly focus on the primary structures of LBPs due to the limitations of techniques and analysis. The primary structure characterization of LBPs covers molecular weight, types and ratios of monosaccharides, positions of glycosidic linkages, anomeric carbon configuration, and branched chains, which influence their biological activities to varying degrees [18,24]. Herein, the research progress on the extraction, purification, and structure of LBPs were summarized below.

### 2.1. Extraction and Purification

The isolation principle of LBPs is to keep the properties of polysaccharides unaltered during the procedure of extraction and purification. Based on this principle, several extraction methods for crude LBPs have been developed, which include cold or hot water extraction, microwave-assisted extraction, enzyme-assisted extraction, ultrasonic-assisted extraction, and supercritical fluid extraction [10,27]. Indeed, water extraction is the most commonly used method to obtain crude LBPs due to its convenient operation and high yield [27,30]. For example, high molecular weight polysaccharides were obtained from dried wolfberries using cold water extraction in a yield of 2–3%, however, the yields of the polysaccharides could be further improved by prolonged high-temperature extraction or enzymatic treatment [30]. Furthermore, it demonstrated that a ratio of water to raw material 31.2, temperature 100 °C, time 5.5 h, and number of extraction 5 were the optimal extraction conditions to obtain LBPs using the Box–Behnken statistical design (predicted yield 23.13%), which was verified by validation experiments (real yield 22.56 ± 1.67%) [31]. Given the excellent solubility of LBPs in water, several scholars have argued that the increased LBPs contain more pectic, cellulose, and hemicellulosic polysaccharides by extended treatments, such as high temperature, enzymatic treatment, and microwave-assisted treatments [31,32].

Generally, the water-soluble extracts using the above extraction methods contain many impurities, such as inorganic salts, pigments, monosaccharides, oligosaccharides, and proteins, which interfere with the structure determination of LBPs. Therefore, effective measures have to be adopted to further purify the above crude LBPs. Hydrogen peroxide, as a chemical reagent, is widely applied in depigmentation and the Sevag method is frequently applied in deproteinization for their simple procedures [33]. Subsequently, the methods for LBP purification can be performed by membrane separation (e.g., ultrafiltration and microfiltration), column chromatography (e.g., gel filtration chromatography, ion-exchange chromatography, affinity chromatography, and cellulose column chromatography), and chemical precipitation (e.g., fractional precipitation with ethanol) alone or in combination [27,33]. Of note, column chromatography is most commonly used in these methods [27]. As we previously reported, five arabinogalactan fractions (LBP1~5) from crude LBPs (extracted by water at room temperature) were separated by DEAE-cellulose chromatography [34]. Afterwards, LbGp1 with a molecular weight of 49.1 kDa was isolated and purified from LBP1 by Sepharedax G-100 column chromatography in yields of 0.018% [22]. Similarly, another five fractions (LRP1, LRP2, LRP3, LRP4, and LRP5) were also isolated from crude *L. ruthenicum* polysaccharides (extraction by 70 °C water) on DEAE-Cellulose-52 anion-exchange column followed by gradient elution in our previous studies [35]. Subsequently, LRGP1 (*Mw* 56.2 kDa) and LRGP3 (*Mw* 75.6 kDa) were further purified on Sephadex G-100 column in yields of 0.003% and 0.008%, respectively [35,36]. Moreover, LBP3b (*Mw* 5 kDa) was purified from crude LBPs extracted with hot water (60 °C) using DEAE-cellulose column and Sephadex G-150 column, which was identified as glucan [24]. In addition, a novel arabinogalactan LBP1A1-1 (*Mw* 45 kDa) was purified from *L. barbarum* on DEAE Sepharose Fast Flow column and Sephacryl S-200 HR column in yields of 0.1% [37]. These studies have indicated that the polysaccharide fractions purified by column chromatography are difficult to investigate for the activities in vivo, as well as the structure–function relationship due to low yield and complex operation. Then, we developed fractional precipitation with 30%, 50%, and 70% (*V*/*V*) ethanol to purify arabinogalactan in yields of 0.38%, which was simpler and more efficient than column chromatography [17].

### 2.2. Structure of LBPs

To date, LBPs have been identified as glycoconjugates that mainly consist of five major structural elements: arabinogalactan, pectin polysaccharide, glucan, xylan, and other heteropolysaccharides [21,22,23,24]. Their hypothetical structure features, such as monosaccharide composition, repeat unit, and molecular weight, were summarized in Table 1. Additionally, the molecular weight of LBPs is highly subject to the origin, cultivar, and extraction method, ranging from 5 kDa to 2300 kDa [10,24,38].

#### 2.2.1. Arabinogalactans

**Figure 1:**
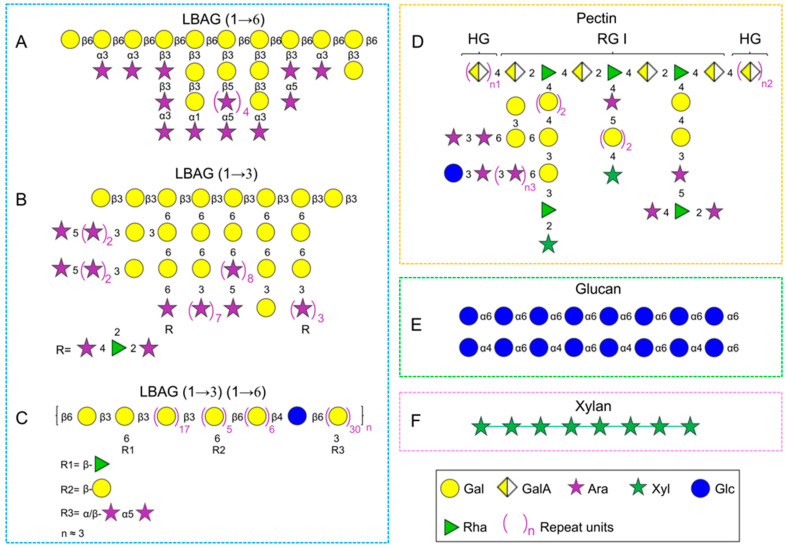
The hypothetical structures of LBPs. The representative arabinogalactan with backbone of (1→6)-linked β-Gal*p* [39] (**A**), (1→3)-linked β-Gal*p* [36] (**B**), (1→3)(1→6)-linked β-Gal*p* [37] (**C**), the typical structure of pectin [23] (**D**), glucan [53] (**E**) and xylan [46] (**F**).

**Figure 2:**
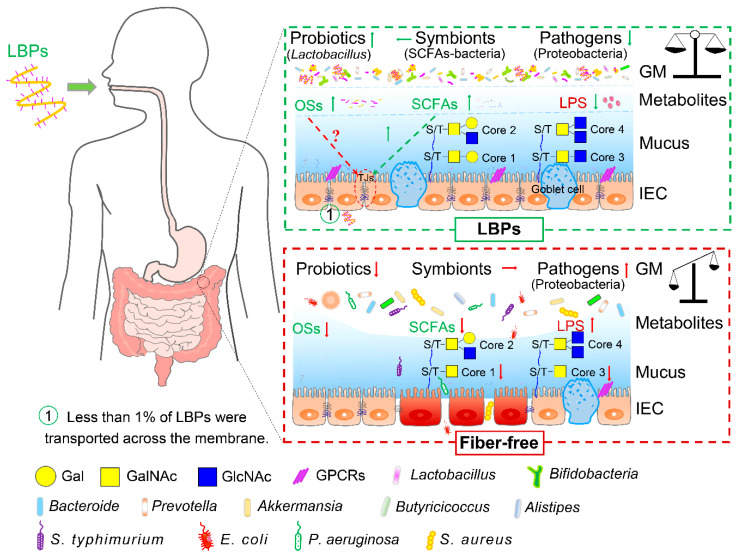
Modulation of LBPs on gut microbiota and intestinal barrier. Green and red arrows refer to the beneficial and detrimental effects, respectively. SCFAs, short-chain fatty acids; GM, gut microbiota; IEC, intestinal epithelial cell; OSs, oligosaccharides derived from LBPs; LPS, lipopolysaccharide; S/T, serine/threonine.

**Figure 3:**
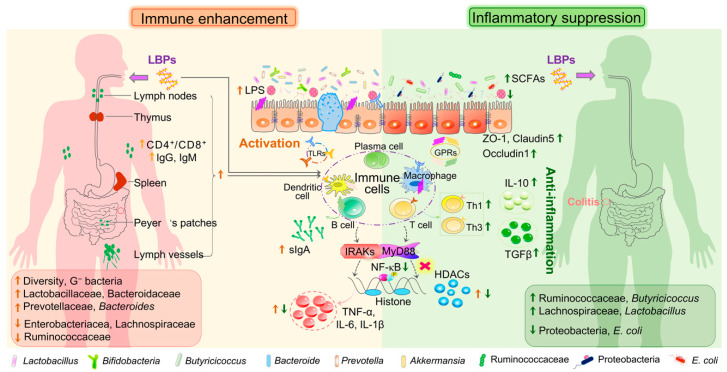
The dual immunomodulatory activity of LBPs in the host. G-bacteria, Gram-negative bacteria; TLRs, Toll-like receptors; GPRs, G protein-coupled receptors; sIgA, secretory immunoglobulin A; TNF-α, tumor necrosis factor α; IL-6/10, interleukin-6/10; IL-1β, interleukin-1β; IRAKs, IL-1, receptor associated kinase; MyD88, myeloid differentiation factor 88; NF-κB, nuclear factor kappa-B; HDACs, histone deacetylase; TGF-β, transforming growth factor-β; Th1/3, T helper type 1/3 cells.

Structural characterization of *L. barbarum* arabinogalactan-protein has been investigated by multiple research groups, and it has been demonstrated that there are a large number of →3,6)-Gal*p*-(1→ residues based on the methylation analysis. The current controversies about its structure are as follows: (1) *L. barbarum* arabinogalactan is composed of →6)-β-Gal*p*-(1→ as the backbone, and large amounts of α/β-Ara*f* as branch chains which substituted at C-3 [22,41,48] (Figure 1A); (2) it is a highly branched polysaccharide with a backbone of →3)-β-Gal*p*-(1→ substituted at C-6 with Ara*f* [40,45] (Figure 1B); (3) the fraction possesses both β-(1→6)-linked Gal*p* and β-(1→3)-linked *Galp* as the backbones with partial substitution at the C-3 site and C-6 site, respectively [37,42] (Figure 1C). The backbone structure of arabinogalactan in LBPs may be different due to diverse origin and various isolation methods. As mentioned above, a combination of ion exchange column and gel filtration column chromatography is commonly employed for the purification of arabinogalactan fraction from *L. barbarum* glycoconjugates; however, it is not suitable for large-scale preparation of arabinogalactan due to complex operation, time-consuming processes, and low yield. Recently, our research team revisited the structure of *L. barbarum* arabinogalactan using a set of chemical methods and analytical techniques, including partial acid hydrolysis, methylation analysis, alkaline degradation, monosaccharide composition analysis, 1H and 13C spectroscopy, and ESI-MS^n^ [39] on the basis of the ethanol precipitation method reported [17]. And the results indicated that it was a highly branched polysaccharide with a backbone of →6)-β-Gal*p*-(1→ and branched chains of →3)-β-Gla*p* (1→, →3)-α-Ara*f*-(1→ and →5)-β-Ara*f*-(1→ substituted at the C3 position, which had an average of 9 branches per 10 sugar backbone units. Additionally, the anti-aging activity of *L. barbarum* arabinogalactan was significantly higher than the backbone fraction (Gal percentage = 91%) obtained by partial acid hydrolysis (0.02 M H_2_SO_4_), indicating that the anti-aging activity was closely relevant to the arabinose branched chains. These results implied that the biological activities of LBPs were considerably influenced by their structures, especially branched chains and spatial configuration [39].

#### 2.2.2. Pectins

Pectins, as a cell wall component of plants, are unique polysaccharides comprising predominantly uronic acids, such as glucuronic acid (GlcA) and galacturonic acid (GalA) [57]. The polysaccharides extracted from *L. barbarum* fruits also contain pectins (Figure 1D). There are mainly three typical structures in pectins: homogalacturonan (HG), rhamnogalacturonan-I (RG-I), and rhamnogalacturonan-II (RG-II) [58,59]. A typical pectic polysaccharide (p-LBP) with a backbone of →4-α-Gal*p*A-(1→ (HG) and a partial region of →4-α-Gal*p*A-(1→ and →2-α-Rha*p*-(1 → (RG-I) was isolated and purified using a series of column chromatographies (e.g., macroporous resin S-8, DEAE column and Sephacryl S400 gel permeation) and analytical techniques (e.g., 1H and 13C spectroscopy) [23]. Another acidic polysaccharide (LBP3a) was also separated from the crude extraction by DEAE-cellulose chromatography, which was identified as HG-type pectin with a backbone of →4)-α-D-GalpA(1→ [53]. HG-type pectin was found in the above studies, perhaps due to the same extraction methods (e.g., hot water) and original place. Besides, the polysaccharides from L. barbarum insoluble cell wall material (CWM) dissolved in the CDTA and Na_2_CO_3_ solutions contained 76.3% and 51.9% uronic acid, respectively. Notably, the fraction extracted by CWM-Na2CO3 may be RG-type pectin, which was supported by the increased level of rhamnose (Rha) [46]. Additionally, one homogeneous polysaccharide (LBP-1, *Mw* 2250 kDa) was purified from crude LBPs using DEAE column, whose structure was identified as pectin with a backbone of α-(1→5)-l-Ara and α-(1→4)-d-GalA, and branched chains of →3)-Man-(1→, →6)-Man-(1→, and T-Man-1(→ [38].

#### 2.2.3. Glucans

Glucans widely exist in the cell walls of various plants and fungi, and there is a small amount in L. barbarum fruits, despite the diversity in conformation and linkages [60]. For instance, LBP1a-1 (Mw 115 kDa) and LBP1a-2 (Mw 94 kDa) were obtained from crude LBPs using DEAE-cellulose and Sephacryl S-400 HR column chromatography, which was identified as glucan with a backbone of →6)-α-d-Glc*p* (1→ [53]. Moreover, a homogenous polysaccharide with a molecular weight of 4.9 kDa was separated from crude LBPs by the DEAE-cellulose column in combination with Sephadex G-150 column and then identified as a β-glucan by monosaccharide composition and 1H/13C NMR analysis [24]. In addition, an α-(1→4) (1→6) glucan (LBPC4) was isolated and purified from crude LBPs using DEAE-cellulose column and Sephadex G-50 column [55].

#### 2.2.4. Xylans

Xylans are the primary hemicellulose component in plant cells, which are mainly found in hardwood (15–30%), softwoods (7–10%), and annual plants (up to 30%) [61]. Additionally, 4 M KOH-soluble fraction isolated from L. barbarum insoluble cell wall material was a xylan instead of xyloglucan, which was supported by the fact that the xylose content was twice that of the glucose [46]. In addition, a β-(1→4) (1→6)-linked heteropolysaccharide (LBPC_2_) was separated from crude LBPs using DEAE-cellulose column and Sephadex G-50 column [55]. Interestingly, it was composed of only Xyl, Rha, and Man in a molar ratio of 8.8:2.3:1.0, so LBPC_2_ was supposed to be a xylan, which needs further confirmation.

#### 2.2.5. Other Polysaccharides

Apart from the above four types, the structural elements of LBPs have been identified as other types from their monosaccharide composition in a few studies. For example, LBP-IV, which is mainly composed of Glc, Ara, and Xyl in a molar ratio of 7.54:3.82:3.44, was separated from crude LBPs on the DEAE-Sephadex A-25 column [56]. Another polysaccharide was isolated from crude LBPs with a macroporous resin S-8 column, which primarily comprised Glc, Man, and Rha in molar ratios of 6.52:2.17:0.81 [26]. These results indicate that LBPs contain other heteropolysaccharides in addition to arabinogalactan, pectin, glucan, and xylan; however, the structures need to be further identified and confirmed. 

## 3. Impact of LBPs on Gut Microbiota and Its Metabolites

### 3.1. Degradation of LBPs by Gut Microbiota

Generally, the polysaccharide chains of LBPs are primarily digested and utilized by gut microbes instead of the host. More concretely, they are hydrolyzed by microbial CAZymes (e.g., GHs and PLs) which are absent in the human genome. For example, the transfer rates of fluorescein isothiocyanate (FITC)-labeled LBP (arabinogalactan-pectin complexes) from basolateral to apical side and vice versa in Caco2 cell monolayer model were 0.98 and 0.92%, respectively, indicating that the transmembrane transport of LBP was extremely limited [62]. Furthermore, LBPs (arabinogalactan type) was not degraded under simulated saliva, gastric, and intestinal conditions, however, it could be utilized and metabolized by gut microbiota based on the consumption of total carbohydrates and promotion of SCFAs after fermentation in vitro [25]. Meanwhile, the above LBPs significantly improved the levels of Bacteroidetes (e.g., *Bacteroides* and *Prevotella*), Firmicutes (e.g., *Lactococcus* and *Faecalibacterium*), and Actinobacteria (e.g., *Bifidobacterium*), perhaps due to the carbohydrate degrading systems of Bacteroidetes (starch utilization system-like systems), Firmicutes, and Actinobacteria (ATP-binding cassette transporters), which implied that LBPs were degraded and utilized by gut microbes in a cooperative manner [8,25]. In addition, an LBP, comprising Glc, Man, Rha, Gal, Ara, Xyl in molar ratios of 6.52:2.17:0.81:0.23:0.18:0.07, markedly promoted the proliferation of the probiotic *Bifidobacterium* and *Lactobacillus* strains by improving the carbon and energy metabolism [26]. Notably, the activity of carbohydrate metabolism enzymes was significantly enhanced by LBP, especially β-galactosidase and lactate dehydrogenase [26]. Actually, microbial culture is an effective method to know and understand the degradation and utilization of LBP by gut microbiota in human health, however, almost none of the existing studies have been applied it to investigate the degradation and utilization mechanism by gut microbiota, perhaps due to the following reasons: (i) the complex structure of LBPs with high branches [27]; (ii) more than 80% of intestinal microbial species are uncultured in vitro [63]; (iii) the specific glycan preference of microbial species [64]; (iv) the cooperation among microbial species [65]. Currently, our research team has explored the microbial degradation of LBPs in pure culture, and two *Bacteroides* species that effectively utilized arabinogalactan from *L. barbarum* have been screened (unpublished data).

### 3.2. Effects on Enteric Pathogens

The dynamic balance of gut microbiota, including the microbial composition and its relative abundance, plays a key role in host intestinal homeostasis [2,66,67]. Numerous studies have demonstrated that the relative abundance of Firmicutes (~64%), Bacteroidetes (~23%), and Proteobacteria (~4.5%) account for over 90% at the phylum level, and any alteration in the microbial proportion tends to the intestinal immune dysregulation and even pathological changes [68,69,70]. Among them, Proteobacteria contains many well-known pathogens such as *Shigellosis*, *Vibrio*, *Salmonella typhimurium*, *Escherichia coli*, *Staphylococcus aureus*, *Helicobacter pylori*, *and Pseudomonas aeruginosa*. LBPs (without chemical characterization) remarkably inhibited the proliferation of pathogenic *E. coli*, *S. typhimurium*, and *S. aureus* in vitro [71,72]. Furthermore, sulfated LBPs with sulfation degrees of 1.5–2.0 could significantly improve antiviral (Newcastle disease virus) activity [73]. Additionally, LBPs with concentrations of 8–20 mg mL^−1^ not only suppressed the growth of *E. coli* in vitro, but reduced cecal *E. coli* in tumor mice [74]. 

Anomalous expansion of Proteobacteria (belonging to Gram-negative bacteria) is the microbial signature of dysbiosis in gut microbiota, and its level can be at least three times higher in inflammation and cancer (14.9%) than that in healthy humans (4.5%) [68]. What is more, Gram-negative bacteria produced more than twice as many pro-inflammatory cytokines IL-6 and IL-8 from human monocytes compared to Gram-positive bacteria [75]. Furthermore, compared to other cell wall constituents of bacteria such as peptidoglycan and teichoic acid, lipopolysaccharide (LPS) is the most efficient endotoxin isolated from bacteria cell walls to induce pro-inflammatory cytokines, which can be recognized by pattern recognition receptors (e.g., TLR4) [76]. The binding of LPS to TLR4 activates the MAPK/NF-κB signaling pathways, and culminates in the generation of pro-inflammatory cytokines (e.g., TNF-α), which is possibly responsible for the intestinal dyshomeostasis caused by pathogenic Proteobacteria, thereby exacerbating inflammation [75,77]. Our previous study reported that LBP-3 (arabinogalactan type) could significantly decrease the abundance of Proteobacteria in DSS-induced colitis mice, especially the pro-inflammatory Enterobacteriaceae, and inhibited the activation of TLR4-MAPK/NF-κB signaling pathways, thereby reducing levels of pro-inflammatory cytokines such as IL-1β and TNF-α [78]. Similarly, LBP (glucan type) remarkably downregulated the level of Proteobacteria, and reduced the LPS/TLR4/NF-κB signaling path in high-fat diet (HFD)-induced nonalcoholic fatty liver disease (NAFLD) in Sprague–Dawley (SD) rats [79]. In addition, supplementation with LBP-W (arabinogalactan type) markedly reversed the relative abundance of Proteobacteria induced by a HFD, turning it toward the normal level [41].

### 3.3. Proliferative Effect on Probiotic Bacteria

The promotion effect of LBPs on microbial richness and diversity is partially attributed to their probiotic function. An appropriate abundance of probiotics such as *Bifidobacterium* and *Lactobacillus* contributes to the maintenance of intestinal epithelial barrier function and the modulation of immune homeostasis by competitive inhibition of pathogens and generation of antimicrobial compounds (e.g., bacteriocins, lactate, and acetate), thereby reducing the inflammation triggered by harmful intestinal bacteria [80]. It has been demonstrated that LBP (without chemical characterization) with concentrations of 12–20 mg mL^−1^ significantly promoted the proliferation of *Lactobacillus* in vitro [74]. Similarly, LBPs mainly composed of Glc, Man, and Rha in molar ratios of 6.52:2.17:0.81 could pronouncedly improve the growth of *B. bifidum*, *B. infantis*, *B. longum*, *B. animalis*, *L. acidophilus*, and *L. plantarum* in vitro [26]. Furthermore, the same type of LBP as the above [26] supported the growth of *L. acidophilus* and *B. longum* with a maximum of 8.23 ± 0.30 (log10 CFU/mL) and 6.34 ± 0.11 (log10 CFU/mL), respectively, in de Man Rogosa Sharpe (MRS) broth; and administration of LBPs to normal mice also markedly improved the relative abundance of probiotic *Lactobacillus*, and enriched sIgA in the colon, thus enhancing the innate immunity [81]. In addition, supplementation with arabinogalactan-type LBP-W (50 mg kg^−1^ d^−1^) not only improved the diversity of gut microbiota but significantly increased the relative abundance of *Lactobacillus* in normal mice and HFD-induced obese mice [41]. More importantly, Ara, Gal, arabino-oligosaccharide, and galacto-oligosaccharide (GOS), as prebiotics, have been indicated to have the proliferative capacity of *Bifidobacterium*, which probably explains why *L. barbarum* arabinogalactans have the prebiotic effect [82,83,84]. Of note, *Bifidobacterium* and *Lactobacillus* (e.g., live combined *Bifidobacterium* and *Lactobacillus* tablets) have been widely used in the clinical treatment of pediatric gastrointestinal diseases (e.g., diarrhea) [85,86]. These research findings suggest LBP is a good potential prebiotic which can boost beneficial bacteria levels, modulate the intestinal microbiota structure, and regulate the intestinal homeostasis of the host.

### 3.4. Impacts on Symbiotic Microbiota

Apart from the above enteric pathogens and probiotics, some commensal microbiota that are well-known glycan utilizers, such as *Akkermansia*, *Prevotella*, *Bacteroide*, Ruminococcaceae, Prevotellaceae, and Bacteroidaceae, can also be enriched by LBPs. These polysaccharide utilizers contain various GHs and PLs which are responsible for the degradation of polysaccharides [7]. We found that the various types of LBPs in similar experimental models could increase the level of *Bacteroides*, such as in the fermentation of arabinogalactan-type [25] and pectin-type [87] LBPs by the human gut microbiota in vitro. Similarly, *Akkermansia*, hailed as an emerging “second generation” probiotic, was also markedly elevated in Kunming mice with a normal diet [81] and in C57BL/6J mice with a normal diet [88] by different LBPs. Furthermore, the relative abundance of Ruminococcaceae, known as secondary bile acids-producing bacteria [89], was also significantly improved by LBPs in normal mice [90] and DSS-induced colitis mice [78]. Unlike the above findings, the levels of SCFA-generating bacteria were altered to various degrees. For example, arabinogalactan-type LBPs could significantly augment the abundance of Bacteroidaceae, Lachnospiraceae, and Ruminococcaceae in cyclophosphamide (CTX)-induced immunocompromised BALB/c mice [91]. In comparison, arabinogalactan-pectin complex WBPPS not only upregulated the levels of Bacteroidaceae and Ruminococcaceae, but also downregulated Rikenellaceae, Marinifilaceae, and *Alistipes* in CTX-induced mice [92]. Furthermore, supplementation with LBP (without chemical characterization) decreased the relative abundance of *A. muciniphila*, *Allobaculum stercoricanis*, *Citrobacter*, *Tannerella*, *Spirochaeta*, and *Parasutterella excrementihominis* in normal C57BL/6J mice fed with a standard diet [88]. Hence, the effects of LBPs on gut microbiota are complicated, perhaps depending on the types of LBPs and animal models. In summary, the beneficial effects of LBPs on the host health may be attributed to the enrichment of probiotics, the decrease of pathogens, and the stabilization of symbiotic bacteria, i.e., its capacity for balancing microbial structure.

### 3.5. Modulation of LBPs on Gut Microbiota-Derived Metabolites

Small molecule metabolites that are generated as intermediate or final products by gut microbiota play a crucial role in the interaction between gut microbiota and the host, which contributes to the modulation of intestinal and systemic immunity. Given that the gut microbiota is a complex microbial community, it is difficult to explain the overall metabolic situation through the metabolism of individual bacteria. SCFAs, secondary bile acids (BAs), and tryptophan are three major microbial metabolites that take part in intestinal epithelial integrity and barrier function [93]. In particular, SCFAs, the main end metabolites produced in LBP fermentation, can regulate host physiology through multiple pathways: (i) lowering the local pH, lubricating the intestinal tract, promoting mucin secretion, and inhibiting the growth of pathogens and their adhesion to intestinal mucosa [94]; (ii) directly suppressing the activity of histone deacetylases (HDACs), which regulate the expression of inflammatory/immune genes, thus reducing the secretion of pro-inflammatory cytokines (e.g., TNF-a) [95]; (iii) activation of G protein-coupled receptors (GPCRs, such as GPR41, GPR43, and GPR109A) on the inner surface of epithelial cells or immune cells, thus triggering immune response in a very rapid manner [96]; (iv) acting as a major energy source for intestinal epithelial cells, promoting epithelial cell proliferation and differentiation, and improving intestinal epithelial barrier function [97]; (v) inhibition of the NF-κB signaling pathway and reduction of oxidative stress, thereby reducing colonic inflammation and even carcinogenesis [98,99]. Although SCFAs include acetate, propionate, *n*-butyrate, *i*-butyrate, *n*-valerate, and *i*-valerate, more than 90% of total SCFAs in the colon are constituted by the first three. Notably, numerous studies have shown that LBPs not only increase the concentrations of SCFAs, but promote the levels of SCFA-producing bacteria, such as acetate-generating *Bifidobacterium*, *Prevotella*, and *Bacteroides* [100,101,102], propionate-producing *Bacteroides*, *Coprococcus,* and *Ruminococcus* [62,78,90,92], and butyrate-producing *Coprococcus* and *Faecalibacterium*. [25,90,103]. In addition, our latest research showed that arabinogalactan-type LBP-3 could reverse the levels of certain specific amino acids (e.g., tryptophan, phenylalanine, lysine, glutamine, homoserine, and leucine) and organic acids, (e.g., kynurenine, 2-isopropylmalic acid, ascorbic acid, gluconic acid, (*S*)-2-hydroxyglutarate, and taurine) disturbed by DSS induction [104]. Moreover, pathway analysis indicated that the pentose phosphate pathway, phenylalanine, tyrosine and tryptophan biosynthesis, and phenylalanine metabolism were also altered by LBP-3 [104]. Additionally, LPS is also considered an intestinal bacterial metabolite, and its level was dramatically reduced by LBPs in HFD/streptozotocin (STZ)-induced diabetes in rats and mice [101,105]. Furthermore, urine metabolomics on an HFD/STZ-induced diabetic rat model revealed that administration of LBPs (glucan type) could enhance the levels of creatinine, 2,2,3-dihydroxybutyric acid, d-galacturonic acid, and citric acid, and reduce methylmalonic acid, benzoic acid, and xylitol, recovering them to normal levels [106]. In addition, supplementation with dietary Goji could decrease the contents of ω-6 polyunsaturated long-chain fatty acids (PUFAs, e.g., linoleic acid and arachidonic acid) and levels of the amino acids (l-valine, l-phenylalanine, l-serine, l-lysine, l-methionine, and l-glutamic acid) which were closely related with intestine inflammation in feces of interleukin (IL)-10-deficient mice [107]. Herein, the modulation of LBPs on gut microbiota and its metabolites in different experimental models (including fermentation of human gut microbiota in vitro) is summarized in Table 2. Considering the complex ecosystem of gut microbes, the alterations in microbial metabolites are probably not solely ascribed to LBPs. So knockout mouse models or isotope tracing methods need to be applied to understand the impacts of LBPs on microbial metabolism. 

More importantly, do the intermediate products (e.g., oligosaccharides) produced from the microbial degradation process of LBPs have benefits to the host? The oligosaccharide fragments liberated by polysaccharide-utilizing members (producers) are potentially available to other species unable to utilize polysaccharides alone (potential recipients) to form the ecological network of polysaccharide utilization among intestinal symbionts [119], which also makes it difficult to obtain active oligosaccharide fragments of LBPs. Furthermore, whether oligosaccharides produced by microbial degradation of polysaccharides have the ability to cross the vascular barrier into the systemic circulation, as well as their functional activities, are not yet known. It has been demonstrated that prebiotic GOS can improve mucosal barrier function by directly stimulating intestinal goblet cells [120,121]. In addition, a portion of oligosaccharides (e.g., GOS, human milk 2’-fucosyllactose, 6’-sialyllactose, and lacto-N-neotetraose) could be absorbed into plasma, thus reaching the systemic circulation [122,123], therefore, we speculated that oligosaccharides from LBP degradation by gut microbes may have access to the systemic circulation. At present, there are few reports about the active oligosaccharide fragments derived from microbial degradation of LBPs. Fortunately, our research group has obtained some oligosaccharides using a single culture of certain *Bacteroides* strains, and these oligosaccharides indeed contain GOS after derivatization with PMP and analysis by RP-HPLC-MS (unpublished data). Thus, more studies are needed to elucidate the degradation mechanism and explore the functions of these intermediate products (e.g., oligosaccharides). 

The effects of LBPs on gut microbiota and its metabolites, as well as intestinal barrier function, are shown in Figure 2. LBPs are predominantly fermented by intestinal microbes to produce favorable metabolites, especially SCFAs, and in turn, they also alter the microbial composition by promoting the proliferation of probiotics, inhibiting the growth of pathogens, and stabilizing commensal bacteria. As described above, the regulatory effect of LBPs on gut microbiota is diverse, owing to different physicochemical properties of LBPs, individual diversity in gut microbes, and even the conditions under different health states. The types of glycosidic linkage, monosaccharide composition, degree of polymerization, and branched chains of LBPs greatly determine the modulation of LBPs on the profiles of gut microbial communities. Hence, how the gut microbes utilize structurally specific LBPs needs to be further investigated using further in vitro and in vivo experiments. 

## 4. Beneficial Health Effects of LBPs Mediated by Gut Microbiota

Numerous studies of the gut microbial genome have so far broadened our understanding of the potential mechanisms underlying human diseases. The gut microbiota can impact host physiological functions and metabolism through promoting energy metabolism and regulating host/diet-derived compounds that alter host metabolic activity [1]. As mentioned in Section 3, the composition of microbial communities can be modulated by LBPs, and in turn, LBPs provide available substrates for fermentation by gut microbes. Dysbiosis of gut microbiota contributes to immune dysregulation, inflammatory responses, and various metabolic disorders in the host [1,66,124,125]. The diversity and composition of gut microbial communities play a crucial role on the maintenance of intestinal homeostasis, allowing the symbiotic fitness between gut microbiota and host immunity. Therefore, the interaction between LBPs and gut microbes is a potentially vital strategy to target host health benefits. The health effects of LBPs have been validated in both mice and human studies; however, the exact underlying mechanisms are still not fully understood. This review will summarize the intervention of LPBs on disease progression based on microbial strategies. 

### 4.1. Impacts of LBPs on Host Immune Modulation

It has been demonstrated that central immune organs (e.g., bone marrow and thymus) and peripheral immune organs (e.g., spleen and intestinal lymph nodes) can be promoted by LBPs, thus enhancing host immunity [126]. However, an overreaction of the immune system can contribute to an uncontrolled inflammatory response and cytokine storm. Administration of LBPs could modulate the development and differentiation of immune cells such as T lymphocytes, B lymphocytes, macrophages, and dendritic cells (DCs), and downregulate the inflammatory immune response, inhibiting the secretion of pro-inflammatory cytokines [127]. Existing studies have primarily focused on the immune regulatory mechanisms of LBPs from a single immune enhancement or inflammatory inhibition instead of a bidirectional immune regulation. Herein, the bidirectional immune regulatory effects of LBPs were summarized and reviewed, of which the intestinal epithelial barrier function in host mucosal immune function has to be mentioned.

#### 4.1.1. Effects on Intestinal Mucosal Barrier Function

The intestinal epithelial barrier was mainly composed of a mucus layer, epithelial cells, and tight junctions (TJs) between epithelial cells [128]. The intestinal mucus layers, acting as the first line of defense against invading and symbiotic microbes, primarily consist of mucin-2 (MUC2) which is a glycoprotein with high-density clusters of *O*-linked glycans [129,130]. Reductions of Core 1 (Galβ1, 3GalNAcα1-Ser/Thr) and Core 3 (GlcNAcβ1, 3GalNAcαSer/Thr) *O*-glycans severely attenuate structural integrity and seriously disrupt intestinal mucosal barrier function, exacerbating microbial degradation of the mucus [131]. Furthermore, the most frequent aberrant glycosylations in inflammatory bowel disease (IBD) patients and animal models are the loss of Core 1 and Core 3 type *O*-glycans [131,132]. Insufficiency of nondigestible polysaccharides contributes to the erosion of the mucus layer by certain gut microbes (e.g., *Akkermansia*) which utilize mucin-type *O*-glycans as alternative nutrients, thus increasing invasion susceptibility of pathogens to intestinal epithelial cells and triggering immune and inflammatory responses [133,134]. Upon intestinal inflammation, a large number of inflammatory cytokines (e.g., IL-1β) and inflammatory mediators (e.g., iNOS) are secreted by intestinal mucosal immune cells (e.g., macrophages), which, in turn, damage intestinal epithelial cells, induce epithelial cell apoptosis, and reduce the expression of TJs, thereby compromising gut mucosal barrier function [135,136]. It has been demonstrated that MUC2-deficiency can lead to the development of spontaneous colitis with histologic damage, thinner mucus layer and increased permeability, which are susceptible to the invasion of epithelial cells by pathogens [137,138]. Our previous study found that the mucus layer got thicker and the expressions of mucin MUC2 and TJs (e.g., Claudin1 and ZO-1) were enhanced after supplementation with LBP-3 (arabinogalactan type) in DSS-induced colitis, thereby improving the intestinal barrier function [78]. In addition, administration of arabinogalactan-type LBPs could significantly elevate the levels of MUC2 and TJs (e.g., Claudin5 and Occludin1) and promote the number of goblet cells in both CTX-treated mice and normal mice [91,108]. Interestingly, one recent study found that sulfated polysaccharides from *Gloiopeltis furcate* could increase the abundances of potential probiotics Muribaculaceae and *Roseburia*, and enhance the levels of complex long-chain mucin *O*-glycans, especially sialylated G29 and G31 that contain eight to ten monosaccharides with two terminal N-acetylneuraminic acid residues, thus improving the intestinal barrier integrity and attenuating DSS-induced colonic mucosal damage [139]. Given that mucin *O*-glycans play a key role in host–microbiome interactions and that the glycan-peptide linkage of arabinogalactan-type LBPs was *O*-glycosidic linkage [22,39,105], arabinogalactan-type LBPs may protect colonic mucus layers by modulating the structure of gut microbiota, and in turn, intact mucin-type *O*-glycans enhance intestinal barrier function and prevent pathogen invasion. However, the protective mechanism of LBPs on mucin-type *O*-glycans needs further exploration.

#### 4.1.2. Immune Enhancing Activity

The gut is the largest immune organ in the body, and it contains differentiated epithelial cells (e.g., enterocytes, goblet cells, and Paneth cells) and intestinal resident-immune cell subsets (e.g., B cells, T cells, DCs, and mesenteric-associated lymph nodes), which account for 70–80% of immune cells. Numerous studies have found that LBPs directly stimulate diverse immune cells or indirectly activate NF-κB signaling pathways through multiple pathways, thus promoting humoral and cellular immunity [10,17,126]. As mentioned in Section 3.1, only less than 1% of LBPs can pass through Caco2 monolayer cells [62], and it is perhaps these very limited LBPs that produce immune benefits, which is beyond the discussion in this review. In addition, Toll-like receptors (TLRs) of intestinal epithelial cells and immune cells (e.g., macrophages and DCs) can recognize pathogenic bacteria and their metabolites (e.g., LPS), which initiates signaling cascades including MyD88 and Interleukin-1 receptor-associated kinase (IRAK) and activates NF-κB signaling pathways, thereby releasing inflammatory mediators and activating the adaptive immune system [140]. Notably, intestinal immune responses are normally tolerant to commensals instead of pathogens in the steady state, and a healthy microbiome dynamically coexists with intestinal immunity in the co-evolution of host and microbe [141]. Consequently, the immune balance can be shaped by the composition of the microbial community.

Many studies have found that the immune benefits of LBPs mediated by gut microbiota on the host are far beyond the gut, and impact the whole systemic immune responses. Arabinogalactan-type LBPS could improve thymus and spleen indexes and alleviate immune organ damage by enriching immune-related Lactobacillaceae, Bacteroidaceae, Verrucomicrobiaceae, and Prevotellaceae, as well as SCFAs in CTX-induced immunosuppressed mice [91]. Meanwhile, LBPs significantly upregulated the production of cytokines (e.g., IL-1β, IL-6, IL-2, IFN-γ, and TNF-α) by elevating the levels of *Bacteroides* and Gram-negative bacteria which contain LPS and activate the TLR4-MyD88-NF-κB signaling pathway [91]. Furthermore, arabinogalactan-pectin WBPPS improved immune function and regulated gut microbiota by increasing the abundances of Ruminococcaceae and Saccharimonadaceae in CTX-treated mice and lowering the levels of Tannerellaceae, Rikenellaceae, and Marinifilaceae, which were closely related to immune traits [92]. Similarly, another arabinogalactan-pectin LBP also exhibited immunoregulatory activity in CTX-induced mice by elevating splenic CD4+/CD8+ T-lymphocyte cell ratios and improving the diversity of gut microbiota, as well as the abundances of bacteria such as Bifidobacteriaceae, Rickenellaceae, and Prevotellaceae [62]. The above studies provide further insight into how LBPs with specific structures improve host immunity through gut microbiota: (i) the above mentioned LBPs exhibited immune enhancing properties, which were mainly composed of Ara, Gal and/or GalA with high branched chains; (ii) all arabinogalactan-type LBPs modulated gut microbiome structure by improving microbial diversity and upregulating the levels of probiotics (e.g., Lactobacillaceae) and commensal bacteria (e.g., Bacteroidaceae and Prevotellaceae), thus recovering them in a state of dynamic balance; (iii) in addition to the direct modulation of gut microbes, SCFAs, the prominent metabolites derived from microbial fermentation of LBPs, can be recognized by GPCRs on the surface of enterocytes or immune cells and involved in host immune response; (iv) LBPs promoted host immunity by directly improving central and peripheral immune organs (e.g., thymus and lymphatic) and indirectly enhancing the production of immune-related cytokines (e.g., IgA, TGF-β1, and TNF-α). Of note, the mechanistic evidence between immune and gut microbiota has been obtained mostly from animal models, and further research is needed to determine whether it can be applied to humans before relevant clinical trials [142].

#### 4.1.3. Suppression on Immune-Inflammation

An appropriate immune response protects the host from pathogenic infection, one overresponse can harm the host, and the inflammatory response is one outcome of an excessive immune reaction [143]. Dysbiosis in gut microbiota contributes to intestinal barrier dysfunction through impairing intestinal epithelial cells and enhancing permeability, and then endotoxins, pathogens, and other unfavorable molecules enter gut lamina propria, which can be recognized by TLR4 on macrophages or CD103+ dendritic cells, thereby triggering intestinal mucosal immune abnormalities [144]. Dietary supplementation with 1% LBP significantly ameliorated colonic mucosal damage, crypt destruction, and inflammatory infiltration, and increased the relative abundance of *Lactobacillus* and *Butyricicoccus* in DSS-induced colitis in wild C57BL/6 mice [145]. However, LBP failed to exert the protective effect against colitis, and fecal butyrate in the LBP group showed no difference compared to DSS treatment in germ-free mice [145]. These results indicated that LBP might alleviate colitis by modulating the composition of gut microbes, especially butyrate-producing bacteria, and gut microbiota seem to be essential for the anti-inflammatory activity of LBPs. In addition, acetate and propionate can inhibit HDAC and GPR43 signaling pathways, which contribute to the promotion of total colonic regulatory T cells (e.g., cTreg, Th1, and Th3) and production of anti-inflammatory cytokines IL-10 and transforming growth factor beta (TGFβ) [99]. Furthermore, our previous study also demonstrated that arabinogalactan-type LBP-3 exhibited an ameliorative effect against DSS-induced colitis by inhibiting the activation of TLR4-MyD88-NF-κB signaling pathways and reshaping the gut microbiota, as well as improving SCFA generation [78]. At present, most research focuses on the immune enhancing activity of LBPs, and less attention is paid to the immunosuppressive effects. Thus, future studies about anti-inflammation and its underlying mechanisms may be needed.

In conclusion, both LBPs and their microbial metabolites, especially SCFAs, demonstrate bidirectional modulation of the immune response. LBPs modulate the host immune response by shaping gut microbiota and regulating the epithelial barrier function, thus establishing a symbiotic relationship of diet–host–microbiota (Figure 3). However, many aspects remain unclear in this symbiotic network: (i) How do LBPs regulate the gut microbes associated with the gut barrier and which bacteria taxa within the microbial community play a decisive role in the gut barrier? (ii) Apart from LBPs and the main metabolite SCFAs, do the intermediate product oligosaccharides have the bidirectional benefit of immunity? (iii) What is the molecular mechanism that causes LBPs to promote mucin secretion? Does it promote the proliferation of goblet cells or reduce the consumption of mucin by gut microbes? (iv) What is the effect of LBPs on the interaction between mucin *O*-glycosylation and gut microbiome? Future studies need to explore the above issues in depth and understand the protection mechanism of LBPs on the intestinal mucosal barrier.

### 4.2. Influence of LBPs on Metabolic Syndrome

**Figure 4:**
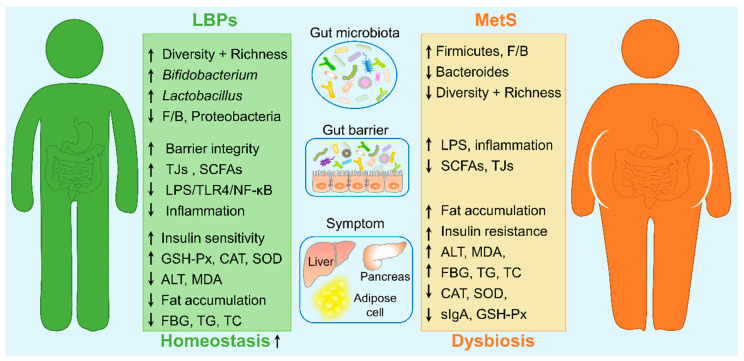
The amelioration of LBPs on metabolic syndrome (MetS). ↑ and ↓ indicate improvement and reduction, respectively. F/B, Firmicutes/Bacteroidetes; TJs, tight junction proteins; GSH-Px, glutathione peroxidase; CAT, catalase; SOD, superoxide dismutase; ALT, alanine aminotransferase; MDA, malondialdehyde; FBG, fasting blood glucose; TG, total cholesterol; TC, triglyceride.

Accumulating evidence has demonstrated that gut microbiota and its metabolites are crucial mediators in host energy metabolism, which participate in the progression of many metabolic diseases such as obesity, type 2 diabetes, and nonalcoholic fatty liver disease [5,146]. Although the etiology of metabolic syndrome (MetS) is still unclear, genetic inheritance, immunity, gut microbiota, and lifestyle may be responsible for the development of MetS [147]. Many studies have demonstrated that LBPs exhibited therapeutic effects on MetS, hence the role and mechanism of LBPs in the treatment of MetS were summarized and reviewed (Figure 4).

#### 4.2.1. Obesity and Diabetes

An expansion of Firmicutes and/or a drop in Bacteroidetes, i.e., an increased F/B ratio, which improves the capacity for the host to efficiently metabolize energy from nutrients, is usually observed in obesity and diabetes in both human and animal models [5,146,148]. Arabinogalactan-type LBP-W could significantly alleviate body weight and fat accumulation in HFD-induced obese mice and ameliorate the concomitant symptoms of hyperlipidemia and hyperglycemia, which are associated with the modulation of gut microbiota, such as improved diversity and richness, and reduced F/B and Proteobacteria (belonging to Gram-negative bacteria) [41]. It has been demonstrated that adipocytes can synthesize inflammatory cytokines such as TNF-a, IL-1β, and IL-6 and then accelerate inflammation in adipose tissue, which contributes to insulin resistance and other metabolic diseases such as type 2 diabetes [149]. Nevertheless, treatment with crude LBPs (without chemical characterization) recovered the gut microbiota dysbiosis by significantly elevating microbial diversity and beneficial bacteria (e.g., *Bifidobacterium*, *Lactobacillus*, and *Alistipes*) as well as their metabolites (e.g., SCFAs), and by reducing F/B ratio and opportunistic pathogens (e.g., *Desulfovibrio*, *Deferribacteres*, *Tenericute,* and *Blautia*) disturbed by STZ, consequently, effectively relieved the symptoms, such as fasting blood glucose (FBG) levels, serum triglycerides (TG), total cholesterol (TC), and plasma LPS levels in STZ-induced diabetes [101]. Interestingly, obesity may be closely related to certain specific bacteria such as *Bifidobacterium*, *Lactobacillus*, and *Akkermansia*, and these microbes are negatively correlated with obesity and type 2 diabetes [150]. Many studies have confirmed that LBPs can promote the proliferation of *Bifidobacterium* and *Lactobacillus* in vitro and in vivo [41,74,81]; however, few studies focus on the modulation of LBPs on *Akkermansia* in obesity [114]. Although these LBPs showed an amelioration effect on MetS, it is still challenging to further explore the potential molecular mechanisms, due to unclear key active components of crude polysaccharides and uncharacterized structures. Of note, a recent study showed that arabinogalactan-type LBPs significantly improved the levels of FBG, glycated hemoglobin, and pancreatic islet β-cell function in HFD/STZ-induced diabetic mice, and simultaneously discovered a key taxon (belonging to genus *Allobaculum*) associated with *n*-butyrate generation [105]. Furthermore, diabetic mice transplanted with LBPs-mediated gut microbiota had similar positive protection toward FBG (a decrease of 16.34%), however, such improvement could be deprived by antibiotics treatment [105]. The above studies suggested that LBPs could serve as a promising option for the treatment of type 2 diabetes based on the modulation of the intestinal microbial ecosystem.

#### 4.2.2. Non-Alcoholic Fatty Liver Disease

The incidence of NAFLD varies from 20% to 30% in the general population and is as high as 75–100% in obesity [151,152]. Many studies have demonstrated that LBPs show protective effects on NAFLD by regulating gut microbiota. For example, intervention with arabinogalactan-pectin type WBPPS effectively improved CTX-induced hepatic tissue damage and oxidative stress by enhancing the activities of glutathione peroxidase (GSH-Px), superoxide dismutase (SOD), and catalase (CAT), and reducing the levels of malondialdehyde (MDA) and alanine aminotransferase (ALT) in the liver, which was closely associated with gut microbial composition, especially Ruminococcaceae, Saccharimonadaceae, and Tannerellaceae [92]. Similarly, administration of arabinogalactan-type LBP-W also could reduce HFD-induced hepatic steatosis, fat accumulation, liver inflammation, and cirrhosis [153]. In addition, the activation of hepatic TLR-4 by gut-derived LPS (via blood circulation) has been implicated in the pathogenesis of diet-induced NAFLD [154]. Meanwhile, glucan-type LBP also could reduce the activation of the LPS/TLR4/NF-κB signaling pathway via downregulating the harmful bacteria Enterococcaceae and its metabolites, LPS, in HFD-induced NAFLD rats, thereby reducing liver inflammation and lesions [79].

An important mechanism for the improvement of LBPs in diet-induced MetS may be that they promote the abundance of SCFA-producing microbiota (e.g., *Lacticigenium*, *Butyricicoccus*, and Lachnospiraceae_NK4A136_group) and simultaneously increase the levels of SCFAs, especially butyric and propionic acid [101,103]. Propionate and butyrate could prevent HFD-induced obesity by modulating free fatty acid receptors 2 and 3 (FFAR2 and FFAR 3) and gut microbes [155]. Furthermore, LBPs could significantly increase the level of *n*-butyrate and suppress the expression of pro-inflammatory cytokines by downregulating the expression of GPR43 and GPR109a and inhibiting the activation of the NF-κB pathway, thereby suppressing systemic obesity and chronic metabolic inflammation [79]. Subsequent studies also confirmed that LBP ameliorated obesity by modulating gut microbiota and SCFA production [41]. More importantly, butyrate and propionate are potent anti-obesity agents, particularly butyrate, playing a key role in the improvement of intestinal permeability and maintenance of gut microbial ecology [94]. In addition, the intestine and liver bidirectionally communicate through the gut–liver axis, which consists of the liver, gut and gut barrier [156]. As described above, LBPs also promote the expression of TJs to maintain gut barrier integrity [78,91,101] to ameliorate MetS. However, crude LBPs were currently employed to explore the protective effect on MetS in most studies, and the potential mechanisms still need further investigation, including: (i) which structural types of LBPs have the positive effect toward MetS, and the structure–activity relationship is unclear; (ii) investigation of the key bacteria and metabolites altered by LBPs is urgent, and the interaction between gut microbiota and its metabolites in MetS is unknown. We propose that future research should focus on the protective mechanism of LBPs with clear structures, provide a new therapeutic strategy for the prevention and treatment of MetS, and lay a foundation for in-depth study of the relationship between the structure and function of LBPs. 

### 4.3. Other Health Benefits of LBPs

Apart from modulation of LBPs-mediated gut microbiota on host immune and MetS, such benefits have also been found in other diseases. For instance, supplementation with LBP (without chemical characterization) significantly improved lung inflammation and pulmonary edema through inhibiting the activation of the NF-κB pathways and cytochrome C in LPS-induced acute respiratory distress syndrome mice [157]. LBPs (without chemical characterization) could alleviate allergic asthma through reducing inflammatory cytokines (e.g., IFN-γ, TNF-α, IL-6, MCP-1, and IL-1β) in plasma and bronchoalveolar lavage fluid, and regulating gut microbiota, especially the improvement of beneficial *Lactobacillus*, *Bifidobacterium*, and *Clostridiales* [102]. In addition, LBE (without chemical characterization) significantly mitigated radiation-induced damage by increasing the potential beneficial bacteria *Akkermansia* and decreasing the relative abundance of harmful Rikenellaceae_RC9_gut_group, as well as modulating the corresponding metabolic pathways (e.g., tryptophan metabolism, indole alkaloids biosynthesis, D-arginine and D-ornithine metabolism, secondary bile acid biosynthesis, and arachidonic acid metabolism) [114].

Recent studies have demonstrated that the gut microbiota is involved in the regulation of emotions, behavior, and cognitive function through the gut–brain axis [158]. For example, LBP (without chemical characterization) may alleviate the emotional damage induced by chronic stress by improving alpha diversity, *Lactobacillus*, Prevotelaceae_ UCG-001, norank_f_Muribaculaceae, and SCFAs, thereby reducing the influence of stress factors on depressive damage in the offspring [113]. In addition, a clinical trial recently indicated that 300 mg d^−1^ LBP (without chemical characterization) could ameliorate depressive symptoms in adolescents with subthreshold depression, and demonstrated good tolerability with no adverse events [159].

## 5. Conclusions and Future Prospects Perspectives

The current review compiles the latest research findings on the isolation, purification, and structural types of LBPs, their modulation impact on gut microbiota, and the associated health benefits on host immunity and MetS. The composition of intestinal microbial communities is crucial for the utilization of LBPs which serve as the fermentation substrate and energy source for gut microbes to regulate gut microbial structure and metabolites. More importantly, the beneficial effects of LBPs on the host differ based on their diverse structural types and seem to be mediated by gut microbiota and its metabolites. In particular, SCFAs have been verified to modulate host immune responses and metabolic homeostasis. Although many studies have suggested that the health effects of LBPs are mediated by gut microbiota, in-depth studies are urgently needed to clarify the molecular mechanisms underlying immunity and MetS, and the following issues remain to be resolved: (i) The biological activities of LBPs have been investigated based on crude polysaccharides in most research, and it is difficult to reveal the molecular mechanism underlying the health effects due to their unclear structures. Meanwhile, another major limitation is a lack of standardization and quality control for the LBP used, which is adverse to subsequent clinical applications. (ii) What are the key gut microbes and enzymes in the degradation and utilization of LBPs? How do LBPs with specific structures shape the gut microbiota? The modulation of LBPs on intestinal microbiota is limited to simply analyzing the microbial diversity and abundance in current studies and the lack of microbial functions. (iii) LBPs could improve the intestinal epithelial barrier by mediating gut microbiota; however, what are molecular mechanisms by which LBPs increase mucin secretion? The interaction between the gut microbiome and mucin *O*-glycans is unclear. Final microbial metabolites, SCFAs, are involved in enhancing intestinal barrier function, regulating host immunity and metabolism, whether the intermediate products oligosaccharides have these benefits is unclear. More studies are needed to determine the metabolite profiles and their impacts on host health after supplementation with LBPs. (iv) LBPs are one of the most studied natural polysaccharides, which have great potential to provide safe and effective treatment for immune and metabolic diseases. However, the underlying mechanism between health effects and LBPs by mediating gut microbiota were mainly investigated in animal models, and large-scale clinical trials are needed to confirm the regulatory effects of LBPs in human immunity and metabolic diseases. In the future, exploring the biological functions of LBPs with diverse clear structures and the precise relationship between chemical structure–gut microbiota–biological activity of LBPs are urgently needed to provide a theoretical basis for how LBPs exert health effects on the human body, and lay a foundation for product development and clinical application.

## Figures and Tables

**Table 1:** Molecular weight, monosaccharide composition, and hypothetic structure of LBPs.

No.	Name	Mw (kDa)	Molar Ratio	Possible Structure of Repeat Unit	Ref.
1	LBGP70-OL	73	Ara:Gal = 1.0:1.0	Backbone: (1→6)-β-Gal*p*; branches: (1→3)-α-Ara*f*, (1→3)-β-Ara*f*, (1→5)-β-Ara*f*, (1→3)-β-Gal*p*	[39]
2	LBP-3	67	Ara:Gal = 1.0:1.6	Backbone: (1→3)-β-Gal*p*; branches: α-(1→3)-Ara*f*, α-(1→4)-Ara*f*, α-(1→5)-Ara*f* and β-(1→6)-Gal*p*	[40]
3	LBP-W	113	Ara:Gal:Rha = 55.6:35.5:8.0	Backbone: (1→6)-β-Gal*p*; branches: (1→3)-α-Rha*p*, (1→3)-β-Gal*p*, (1→3)-α-Ara*f*, (1→5)-α-Ara*f*	[41]
4	LBP1A1-1	45	Ara:Gal:Glc:Rha = 47.8:49.8:1.4:1.2	Backbone: (1→3)-β-Gal*p*, (1→6)-β-Gal*p* and (1→4)-β-Glc*p*; branches: (1→6)-β-Gal*p* on C-3 or (1→3)-β-Gal*p* on C-6.	[37]
5	LBP1B-S-2	80	Ara:Gal:Glc:Rha = 53.6:39.4:4.0:3.1	Backbone: (1→3)-β-Gal*p* and (1→6)-β-Gal*p*; branches: (1→4)-β-Glc*p*A, (1→6)-β-Gal*p*, (1→5)-α-Ara*f*	[42]
6	LBLP5-A-OL1	71	Ara:Gal:Rha = 1.0:1.2:0.1	Backbone: (1→3)-linked Gal*p*; branches: (1→6)-linked Gal*p*, (1→3)-linked Gal*p*, (1→3)-linked Ara*f*, (1→4)-linked Ara*f*, (1→5)-linked Ara*f*, and (1→2,4)-linked Rha*f*	[43]
7	LBPA	470	Ara:Gal:GlcA:Rha = 9.2:6.6:1.0:0.9	Backbone: (1→6)-β-d-Gal*p*; branches: (1→3)-α-Ara*f*, (1→5)-α-Ara*f*, (1→6)-β-Glc*p*A, (1→4)-α-Rha*p*	[44]
8	LbGp1	49	Ara:Gal = 5.6:1.0	Backbone: (1→ 6)-β-Gal*p*; branches: (1→2)-linked Ara*f*, (1→3)-linked-Ara*f*, (1→3)-linked Gal*p*, and (1→4)-linked Gal*p*	[22]
9	LRGP3	76	Ara:Gal:Rha = 14.9:10.4:1.0	Backbone: (1→3)-β-d-Gal*p*; branches: (1→5)-α-Ara*f*, (1→2)-α-Ara*f*, (1→6)-β-Gal*p*, (1→3)-Gal*p*, and (1→2,4)-α-Rha*p*	[36]
10	LRGP1	56	Ara:Gal:Glc:Rha:Man:Xyl = 10.7:10.4:1.0:0.7:0.7:0.3	Backbone: (1→3)-linked Gal; branches: (1→2)-linked Ara, (1→5)-linked Ara, (1→3)-linked Gal, (1→4)-linked Gal, (1→6)-linked Gal, and (1→2)-linked Rha	[35]
11	AGPs	ND	Gal:Ara:GlcA:Rha:GalA 44.3: 42.9:7.0 3.3:2.4	Backbone: (1→3)-β-d-Gal*p*; branches: (1→5)-α-Ara*f*, T-α-Ara*f*, T*-*β-Ara*f*, T-α-Rha*p*, and T-β-Glc*p*A	[45]
12	WSP1	ND	Ara:Gal:Glc:HexA:Xyl:Rha:Man = 51.4:25.9:7.3:7.4:4.8:1.6:1.2:	Backbone: (1→3)-Gal*p*; branches: Ara*f* and Gal*p* substituted on O-6	[46]
13	LbGp4	215	Gal:Ara:Rha:Glc =2.5:1.5:0.43:0.23	Backbone: (1→4)-β-Gal; branches: (1→3)-β-Gal with T-α-Ara-(1→ and T-β-Rha-(1→	[47]
14	LbGp2	68	Ara:Gal = 4:5	Backbone: (1→6)-β-Gal*p*; branches: (1→3)-β-Ara*f* and (1→3)-β-Gal*p* with T-α-Ara*f*-(1→	[48]
15	LbGp4-OL	181	Ara:Gal:Rha = 1.3:1.0:0.1	Backbone: (1→4)-linked Gal*p*; branches: (1→3)-β-Gal*p*, (1→3)-α-Rha*p*, (1→3)-β-Ara*f*, (1→5)-β-Ara*f*	[49]
16	LbGp1-OL	40	Ara:Gal = 1:1	Backbone: (1→6)-β-Gal*p*; branches: (1→3)-β-Gal*p*, (1→3)-β-Ara*f*, and T-α-Ara*f*-(1→	[50]
17	LbGp3	93	Ara:Gal = 1:1	Backbone: (1→4)-β-Gal*p*; branches: (1→3)-β-Ara*f* and (1→3)-α-Gal*p* with T-α-Ara*f*-(1→	[51]
18	LBPA3	66	Ara:Gal = 1.2:1.0	Heteropolysaccharide with (1→4), (1→6)-β-linkage.	[52]
19	p-LBP	64	GalA:Ara:Gal:Rha:Glc:GlcA:Xyl: Fuc = 137.0:54.8:23.0:6.4:4.1:3.4:3.0: 1.0	Backbone: (1→4)-α-Gal*p*A; branches: (1→2)-α-Rha*p* on C4 and (1→3)-β-Gal*p* on C-6	[23]
20	WSP2	ND	GalA:Ara:Gal:Xyl:Glc:Rha = 76.0:12.3:6.3:1.8:1.5:1.4	(1→4)-Gal*p*A	[46]
21	LBP-1	2250	GalA:Ara:Man:Rha:Gal:Xyl = 8.2:7.9:3.0:1.0:0.7:0.4	Backbone: α-(1→5)-l-Ara and α-(1→4)-d-GalA; branches: →1)-Man-(3→6) and T-Man-(1→	[38]
22	LBP3a-1/2	103/82	GalA	α-(1→4)-GalA	[53]
23	LBP3b	5	Glc:Man:Rha:Xyl:Gal = 28.1:5.5:5.1:1.7:1.0	β-glucan	[24]
24	LBP_3p_	157	Glc:Man:Xyl:Rha:Ara:Gal = 2.1:2.0:1.8:1.3:1.1:1.0	β-d-Glc linkage	[54]
25	LBP1a-1/2	115/94	Glc	α-(1→6)-d-glucan	[53]
26	LBPC_4_	10	Glc	α-(1→4) (1→6)-glucan	[55]
27	LBPC_2_	12	Xyl:Rha:Man = 8.8:2.3:1.0	Heteropolysaccharide with (1→4) (1→6)-β-linkage	[55]
28	CWM-4M KOH	ND	Xyl:Ara:HexA:Glc:Gal:Man:Rha = 31.9:19.1:18.0:15.1:10.1:4.8:1.8	(1→4) xylan	[46]
29	LBP-IV	420	Glc:Ara:Xyl:Rha:Gal = 7.5:3.8:3.4:1.6:1.0	Backbone: α/β-Ara/Glc; branches: T-Rha	[56]
30	LBP	ND	Glc:Man:Rha:Gal:Ara:Xyl = 6.5:2.2:0.8:0.2:0.2:0.1	ND	[26]

Abbreviations: Gal, galactose; Glc, glucose; Rha, rhamnose; Man, mannose; Ara, arabinose; Xyl, xylose; GalA, galacturonic acid; GlcA, glucuronic acid; HexA, hexuronic acid; ND: not detect. Among the above numbers, No.1–No.18 belong to arabinogalactan; No.19–No.22 belong to pectin type; No.23–No.26 belong to glucan type; No.27–No.28 belong to xylan type; No.29–No.30 belong to other type.

**Table 2:** The modulation effects of LBPs on the gut microbiota and metabolites in different models.

LBPs	Models	Dosage, Duration and Methods	Diversity and Composition of Gut Microbiota	Metabolites	Ref.
LBPs	Chow diet fed in male BALB/c mice	200 mg kg^−1^, 14 weeks, 16S rRNA	↑*Turicibacter*, *Clostridium*, *Barnesiella*, *Prevotella*, *Lactobacillus*; → Diversity, richness	↑Acetate, propionate, butyrate, total SCFAs	[108]
LBP-W	Standard diet fed in male C57BL/6 mice	50 mg kg^−1^, 12 weeks, 16S rRNA	↑*Lactobacillus*; ↓Richness, F/B; → Diversity, Proteobacteria	→Acetate, ropionate, butyrate	[41]
LBP	Normal chow in male C57BL/6J mice	3%, 10 weeks, 16S rDNA	↑Diversity, richness, Ruminococcaceae_UCG-014, *Anaerotruncus*, *Odoribacter*, *Coprococcus*_1, *Candidatus_Saccharimonas*, *Akkermansia*; ↓*Mucispirillum*, *Helicobacter*, *Bacteroides*, *Ruminiclostridium*_9, *Alistipes*	↑Acetate, propionate, butyrate, valerate, total SCFAs	[90]
LBP	Standard diet fed in C57BL/6J mice	750 mg kg^−1^, 15 days, 16S rRNA, ERIC-PCR	↑Diversity, Clostridium, *Lachnoclostridium xylanolyticum*, *Lactobacillus reuter*i; ↓*Barnesiella*, *Bacteroides acidifaciens*, *Akkermansia muciniphila*, *Allobaculum stercoricanis*, *Citrobacter*, *Tannerella*, *Spirochaeta*, *Parasutterella excrementihominis*, *Anaeroplasma bactoclasticum*	↑Serum propionate, butyrate; →valerate, *i*-butyrate	[88]
LBP	Basal diets in weaned piglets	4 g kg^−1^, 14 d, qPCR	↑*Bifidobacterium*, *Lactobacillus*, *Bacteroidetes*; ↓*Escherichia coli*, Firmicutes	ND	[100]
LBP	Standard diet fed in Kunming mice	0.1 mL 10 g^−1^, 14 days, 16S rRNA	↑Firmicutes, Proteobacteria, *Akkermansia*, *Lactobacillus*, Prevotellaceae	ND	[81]
LBP-3	DSS-induced chronic colitis in male C57BL/6J mice	100 mg kg^−1^ d^−1^, 16S rDNA	↑Diversity, richness, Bacteroidetes, Muribaculaceae, Rikenellaceae, Prevotellaceae, Lachnospiraceae, Ruminococcaceae; ↓Proteobacteria, *Helicobacter*, Peptostreptococcaceae, Enterobacteriaceae, Streptococcaceae, Burkholderiaceae; →Firmicutes	↑Acetate, propionate, valerate, total SCFAs; →butyrate	[78]
FGJ	DSS-induced UC in C57BL/6 mice	20 mL kg^−1^ d^−1^, 30 d, 16S rRNA	↑Bacteroidetes, Epsilonbacteraeota, Muribaculaceae, Ruminococcaceae; ↓Firmicutes, Lachnospiraceae, *Odoribacter*	ND	[109]
LBP	CTX-induced immunosuppression in female Kunming mice	100 mg·kg^−1^, 11 days, 16S rRNA	↑Diversity, richness, Firmicutes, Lactobacillaceae, Bacteroidaceae, Prevotellaceae; ↓Lachnospiraceae, Ruminococcaceae, Enterobacteriaceae	ND	[62]
WBPPS	CTX-induced immunosuppression in male BALB/c mice	100/300 mg kg^−1^ d^−1^, 16S rRNA	↑Bacteroidetes, Ruminococcaceae; ↓Tannerellaceae, Rikenellaceae, Marinifilaceae, *Alistipes*, *Helicobacter*, *Rikenella*; →diversity, richness, Saccharimonadaceae	ND	[92]
LBPS	CTX-induced immunosuppression in male BALB/c mice	50, 100, 200 mg kg^−1^ d^−1^, 9 days, 16S rRNA	↑Bacteroidaceae, Lachnospiraceae, Lactobacillaceae, Ruminococcaceae, Porphyromonadaceae, Deferribacteraceae, Verrucomicrobiaceae; ↓Firmicutes, Proteobacteria; →Diversity, richness, Prevotellaceae	↑Acetate, propionate, butyrate, total acids; →*i*-butyrate, valerate	[91]
LBP-W	HFD-induced obesity in male C57BL/6 mice	50 mg kg^−1^, 12 weeks, 16S rRNA	↑Diversity, richness, *Lactobacillus*; ↓F/B, Proteobacteria	↑Acetate, propionate, butyrate	[41]
LBPs	HFD-induced obesity in male ICR mice	0.2%, 10 weeks, 16S rRNA	↑Diversity, Bacteroidetes, *Lacticigenium*, *Butyricicoccus*,*Bacteroides, Faecalibaculum, Bifidobacterium*; ↓Firmicutes, F/B; →richness	↑Butyrate; →acetate, propionate	[103]
LBP	HFD induced obesity in male SD rats	90 mg kg^−1^,12 weeks, 16S rRNA	↑Diversity; ↓F/B	↑Serotonin, 3-methyluridine, PE (22:5n6/0:0), PE (20:3/0:0), PE (P-18:0/0:0)	[110]
LBPs	HFD/STZ-induced diabetes in male C57BL/6 mice	200 mg kg^−1^ d^−1^, 12 weeks, 16S rRNA	↑Bacteroidetes, Actinobacteria, OTU5, OTU538, OTU756; ↓Firmicutes	↑Butyrate; ↓LPS; →acetate, propionate, valerate and total SCFAs	[105]
LBO	HFD and STZ-induced diabetes in male C57BL/6 mice	200 mg kg^−1^, 4 weeks, 16S rRNA	↑Diversity, richness, Bacteroidetes, Prevotellaceae, *Bacteroides*, *Akkermansia*; ↓Lachnospiraceae	↑Proline, serine, leucine, lactose; ↓capric acid, dodecanoic acid	[111]
LLB	HFD and STZ induced T2DM in rats	2.08 g kg^−1^, 4 weeks, 16S rDNA	↓Marvinbryantia, Blautia, Parasutterella, Ruminococcus_1, and Coprococcus_2, Prevotellaceae_NK3B31_group,	↑Malonic acid, hippuric acid; ↓neriantogenin, niacinamide, histidinal homovanillin, xanthosine	[112]
LBP	STZ-induced diabetes in SD rats	400 mg kg^−1^, 8 weeks, 16S rRNA	↑Diversity, richness, Bacteroidetes, *Bifidobacterium*, *Lactobacillus*, *Alistipes*, *Cyanobacteria*; ↓F/B, Firmicutes, *Deferribacteres*, *Tenericutes*, *Blautia*, *Desulfovibrio*	↓LPS; ↑acetate, propionate, butyrate, valerate	[101]
LBP	HFD-induced NAFLD in SD rats	50 mg kg^−1^, 8 weeks, 16S rDNA	↑Deferribacteraceae; ↓Enterococcaceae	↑Acetate, n-butyrate, valerate; →propionate, i-valerate, caproate	[79]
LBP	Prenatal chronic stress in SD rats and offspring	40 mg kg^−1^, 2 weeks, 16S rRNA	Mothers: ↑diversity, richness, Firmicutes; ↓Bacteroidetes, Muribaculaceae, Prevotellaceae; offspring: ↑diversity, Firmicutes, Muribaculaceae; ↓Bacteroidetes, Prevotelaceae, *Turicibacter*	Offspring: ↑SCFA; ↓5-HT, GABA	[113]
LBE	TBI-induced radiation in male C57BL/6 mice	3.0 g kg^−1^, 28 days, 16S rRNA	↑F/B, Clostridium_sensu_stricto_1, *Faecalibaculum*, *Akkermansia*, *Turicibacter*; ↓*Muribaculum*, Rikenellaceae_RC9_gut_group	↑Tetrahydrofolic acid, arginyl-tryptophan, N-acetyl-l-phenylalanine, N-ornithyl-l-taurine; ↓4-pyridoxic acid, methyl-pyrazine	[114]
LBP	OVA-induced asthma in female C57BL/6 mice	100 mg kg^−1^, 4 weeks, 16S rRNA	↑Diversity, richness, *Lactobacillus*, *Bifidobacterium*, *Clostridiales*; ↓Firmicutes, Actinobacteria, *Alistipes*	ND	[102]
LBP	HFD-induced myocardial injury in C57BL/6J male mice	100 mg·kg^−1^, 8 weeks, 16S rRNA	↑*Parabacteroides*, *Gordonibacter*, *Anaerostipes*, *Blautia*, *Hungatella*, *Marvinbryantia*	↑l-Ascorbate, daidzein, hexanoic acid, cholic acid, riboflavin; ↓d-lactate, isomaltose, isoleucine, tryptophan, maltopentaose,	[115]
LBP	Ethanol-induced gastric ulcer in male SD rats	100 mg kg^−1^, 1 week, 16S rRNA	↑Bacillaceae; →Diversity, richness, F/B	ND	[116]
CA, SC	Human gut microbiota in vitro	10 mg 1.8 mL^−1^, 24 h, qPCR	↑*Bifidobacteria*, *Lactobacilli*, *Bacteroides*;→*E. coli*, *total bacteria*	↑Acetate, propionate, butyrate, valerate, total SCFAs	[87]
LBE/LBP	Single culture by *A. muciniphila* in vitro	4/1 mg mL^−1^,24 h, OD_600 nm_	↑*Akkermansia muciniphila*	ND	[114]
DGBE-3	Single culture by *Lactobacillus* and *Bifidobacterium* in vitro	0.1%, 24 h, viable counts	↑*L. acidophilus*, *B.longum*, *B. lactis*, *L. rhamnosus*, *L. casei*	↑Organic acids	[117]
LBPS	Human gut microbiota in vitro	10% in medium, 24 h, 16S rRNA	↑Diversity, richness, *Bacteroides*, *Lactococcus*, *Bifidobacterium*, *Phascolarctobacterium*, *Prevotella, Faecalibacterium*,*Collinsella*	↑Acetate, propionate, butyrate, valerate, total SCFAs; →lactic acid	[25]
WBPPS	Human gut microbiota in vitro	10 mg 1 mL^−1^, 24 h, 16S rDNA	↑*Prevotella*, *Dialister*, *Faecalibacterium*, *Megamonas*, *Alloprevotella*; ↓richness, F/B, *Bacteroides*, *Clostridium XlVa*, *Parabacteroides*, *Escherichia/Shigella*, *Phascolarctobacterium*, *Parasutterella*, *Clostridium sensu stricto Fusobacterium*; →diversity	↑Lactate, acetate, propionate, *n*-butyrate, *n*/*i*-valerate, total SCFAs; →*i*-butyrate	[118]
LBP	Single culture by *Bifidobacterium* and *Lactobacillus* in vitro	5 g L^−1^ in MRS medium, 16 h, viable counts	↑*B. animalis* BY-02, *L. plantarum* LP39, *B. bifidum* Bb-02, *B. longum subsp. longum* A6, *B. longum subsp. infantis* Bi-26; →*L. acidophilus* NCFM, *B. animalis* subsp. lactis Bi-04	ND	[26]

Abbreviations: HFD, high-fat diet; F/B, Firmicutes/Bacteroidetes ratio; STZ, streptozotocin; SD, Sprague–Dawley; OVA, ovalbumin; ERIC, enterobacterial repetitive intergenic consensus; DSS, dextran sulfate; CTX, cyclophosphamide; NAFLD, nonalcoholic fatty liver disease; qPCR, quantitative polymerase chain reaction; DSS, dextran sulphate sodium; SCFAs, short-chain fatty acids; 5-HT, 5-hydroxytryptamine; GABA, γ -aminobutyric acid; TBI, total body irradiation; UC, ulcerative colitis; T2DM, type 2 diabetic mellitus; ND, not detect; ↑, increase; ↓, decrease; →, no significant difference.

## Data Availability

No new data were created or analyzed in this study. Data sharing is not applicable to this article.

## References

[1] Tremaroli V., Bäckhed F. (2012). Functional interactions between the gut microbiota and host metabolism. Nature.

[2] Clemente J.C., Ursell L.K., Parfrey L.W., Knight R. (2012). The impact of the gut microbiota on human health: An integrative view. Cell.

[3] Clarke G., Stilling R.M., Kennedy P.J., Stanton C., Cryan J.F., Dinan T.G. (2014). Minireview: Gut microbiota: The neglected endocrine organ. Mol. Endocrinol..

[4] Jandhyala S.M., Talukdar R., Subramanyam C., Vuyyuru H., Sasikala M., Nageshwar Reddy D. (2015). Role of the normal gut microbiota. World J. Gastroenterol..

[5] Shi Q., Dai L., Zhao Q., Zhang X. (2022). A review on the effect of gut microbiota on metabolic diseases. Arch. Microbiol..

[6] Thursby E., Juge N. (2017). Introduction to the human gut microbiota. Biochem. J..

[7] Tannock G.W. (2021). Modulating the gut microbiota of humans by dietary intervention with plant glycans. Appl. Environ. Microbiol..

[8] Koropatkin N.M., Cameron E.A., Martens E.C. (2012). How glycan metabolism shapes the human gut microbiota. Nat. Rev. Microbiol..

[9] Koh A., De Vadder F., Kovatcheva-Datchary P., Bäckhed F. (2016). From dietary fiber to host physiology: Short-chain fatty acids as key bacterial metabolites. Cell.

[10] Tian X., Liang T., Liu Y., Ding G., Zhang F., Ma Z. (2019). Extraction, structural characterization, and biological functions of *Lycium barbarum* polysaccharides: A review. Biomolecules.

[11] Fukuda T., Yokoyama J., Ohashi H. (2001). Phylogeny and biogeography of the genus *Lycium* (Solanaceae): Inferences from chloroplast DNA sequences. Mol. Phylogenet. Evol..

[12] Jiapaer R., Sun Y., Zhong L., Shen Y., Ye X. (2013). A review of phytochemical composition and bio-active of *Lycium barbarum* fruit (Goji). Zhongguo Shipin Xuebao.

[13] Pharmacopoeia Commission of the Ministry of Health of the People’s Republic of China (2020). Pharmacopoeia of the People’s Republic of China.

[14] Ming M., Guanhua L., Zhanhai Y., Guang C., Xuan Z. (2009). Effect of the *Lycium barbarum* polysaccharides administration on blood lipid metabolism and oxidative stress of mice fed high-fat diet in vivo. Food Chem..

[15] Tang R., Chen X., Dang T., Deng Y., Zou Z., Liu Q., Gong G., Song S., Ma F., Huang L. (2019). *Lycium barbarum* polysaccharides extend the mean lifespan of *Drosophila melanogaster*. Food Funct..

[16] Gong G., Liu Q., Deng Y., Dang T., Dai W., Liu T., Liu Y., Sun J., Wang L., Liu Y. (2020). Arabinogalactan derived from *Lycium barbarum* fruit inhibits cancer cell growth via cell cycle arrest and apoptosis. Int. J. Biol. Macromol..

[17] Gong G., Dang T., Deng Y., Han J., Zou Z., Jing S., Zhang Y., Liu Q., Huang L., Wang Z. (2018). Physicochemical properties and biological activities of polysaccharides from *Lycium barbarum* prepared by fractional precipitation. Int. J. Biol. Macromol..

[18] Cheng J., Zhou Z.W., Sheng H.P., He L.J., Fan X.W., He Z.X., Sun T., Zhang X., Zhao R.J., Gu L. (2015). An evidence-based update on the pharmacological activities and possible molecular targets of *Lycium barbarum* polysaccharides. Drug Des. Dev. Ther..

[19] Chang C.C., So K.F. (2015). Lycium Barbarum and Human Health.

[20] Gao Z., Ali Z., Khan I.A. (2008). Glycerogalactolipids from the fruit of *Lycium barbarum*. Phytochemistry.

[21] Jin M., Huang Q., Zhao K., Shang P. (2013). Biological activities and potential health benefit effects of polysaccharides isolated from *Lycium barbarum* L.. Int. J. Biol. Macromol..

[22] Wang Z., Liu Y., Sun Y., Mou Q., Wang B., Zhang Y., Huang L. (2014). Structural characterization of LbGp1 from the fruits of *Lycium barbarum* L.. Food Chem..

[23] Liu W., Liu Y., Zhu R., Yu J., Lu W., Pan C., Yao W., Gao X. (2016). Structure characterization, chemical and enzymatic degradation, and chain conformation of an acidic polysaccharide from *Lycium barbarum* L.. Carbohydr. Polym..

[24] Tang H.-L., Chen C., Wang S.-K., Sun G.-J. (2015). Biochemical analysis and hypoglycemic activity of a polysaccharide isolated from the fruit of *Lycium barbarum* L.. Int. J. Biol. Macromol..

[25] Ding Y., Yan Y., Peng Y., Chen D., Mi J., Lu L., Luo Q., Li X., Zeng X., Cao Y. (2019). In vitro digestion under simulated saliva, gastric and small intestinal conditions and fermentation by human gut microbiota of polysaccharides from the fruits of *Lycium barbarum*. Int. J. Biol. Macromol..

[26] Zhou F., Jiang X., Wang T., Zhang B., Zhao H. (2018). *Lycium barbarum* polysaccharide (LBP): A novel prebiotics candidate for *Bifidobacterium* and *Lactobacillus*. Front. Microbiol..

[27] Masci A., Carradori S., Casadei M.A., Paolicelli P., Petralito S., Ragno R., Cesa S. (2018). *Lycium barbarum* polysaccharides: Extraction, purification, structural characterisation and evidence about hypoglycaemic and hypolipidaemic effects. A review. Food Chem..

[28] Wu D., Guo H., Lin S., Lam S., Zhao L., Lin D., Qin W. (2018). Review of the structural characterization, quality evaluation, and industrial application of *Lycium barbarum* polysaccharides. Trends Food Sci. Technol..

[29] Amagase H., Farnsworth N.R. (2011). A review of botanical characteristics, phytochemistry, clinical relevance in efficacy and safety of *Lycium barbarum* fruit (Goji). Food Res. Int..

[30] Bucheli P., Gao Q., Redgwell R., Vidal K., Wang J., Zhang W., Benzie I.F.F., Wachtel-Galor S. (2011). Biomolecular and clinical aspects of Chinese wolfberry. Herbal Medicine: Biomolecular and Clinical Aspects.

[31] Yin G., Dang Y. (2008). Optimization of extraction technology of the *Lycium barbarum* polysaccharides by Box–Behnken statistical design. Carbohydr. Polym..

[32] Zhang M., Zhang S. (2007). Study on structure of *Lycium barbarum* L. polysaccharide. Food Res. Dev..

[33] Ren Y., Bai Y., Zhang Z., Cai W., Del Rio Flores A. (2019). The preparation and structure analysis methods of natural polysaccharides of plants and fungi: A review of recent development. Molecules.

[34] Huang L., Lin Y., Tian G., Ji G. (1998). Isolation, purification and physico-chemical properties of immunoactive constituents from the fruit of *Lycium barbarum* L.. Yao Xue Xue Bao.

[35] Peng Q., Lv X., Xu Q., Li Y., Huang L., Du Y. (2012). Isolation and structural characterization of the polysaccharide LRGP1 from *Lycium ruthenicum*. Carbohydr. Polym..

[36] Peng Q., Song J., Lv X., Wang Z., Huang L., Du Y. (2012). Structural characterization of an arabinogalactan-protein from the fruits of *Lycium ruthenicum*. J. Agric. Food Chem..

[37] Zhou L., Liao W., Chen X., Yue H., Li S., Ding K. (2018). An arabinogalactan from fruits of *Lycium barbarum* L. inhibits production and aggregation of Aβ(42). Carbohydr. Polym..

[38] Zou S., Zhang X., Yao W., Niu Y., Gao X. (2010). Structure characterization and hypoglycemic activity of a polysaccharide isolated from the fruit of *Lycium barbarum* L.. Carbohydr. Polym..

[39] Huang W., Zhao M., Wang X., Tian Y., Wang C., Sun J., Wang Z., Gong G., Huang L. (2022). Revisiting the structure of arabinogalactan from *Lycium barbarum* and the impact of its side chain on anti-ageing activity. Carbohydr. Polym..

[40] Wu J., Chen T., Wan F., Wang J., Li X., Li W., Ma L. (2021). Structural characterization of a polysaccharide from *Lycium barbarum* and its neuroprotective effect against β-amyloid peptide neurotoxicity. Int. J. Biol. Macromol..

[41] Yang Y., Chang Y., Wu Y., Liu H., Liu Q., Kang Z., Wu M., Yin H., Duan J. (2021). A homogeneous polysaccharide from *Lycium barbarum*: Structural characterizations, anti-obesity effects and impacts on gut microbiota. Int. J. Biol. Macromol..

[42] Zhou L., Huang L., Yue H., Ding K. (2018). Structure analysis of a heteropolysaccharide from fruits of *Lycium barbarum* L. and anti-angiogenic activity of its sulfated derivative. Int. J. Biol. Macromol..

[43] Gong G., Fan J., Sun Y., Wu Y., Liu Y., Sun W., Zhang Y., Wang Z. (2016). Isolation, structural characterization, and antioxidativity of polysaccharide LBLP5-A from *Lycium barbarum* leaves. Process Biochem..

[44] Yuan Y., Wang Y.-B., Jiang Y., Prasad K.N., Yang J., Qu H., Wang Y., Jia Y., Mo H., Yang B. (2016). Structure identification of a polysaccharide purified from *Lycium barbarium* fruit. Int. J. Biol. Macromol..

[45] Redgwell R.J., Curti D., Wang J., Dobruchowska J.M., Gerwig G.J., Kamerling J.P., Bucheli P. (2011). Cell wall polysaccharides of Chinese Wolfberry (*Lycium barbarum*): Part 2. Characterisation of arabinogalactan-proteins. Carbohydr. Polym..

[46] Redgwell R.J., Curti D., Wang J., Dobruchowska J.M., Gerwig G.J., Kamerling J.P., Bucheli P. (2011). Cell wall polysaccharides of Chinese Wolfberry (*Lycium barbarum*): Part 1. Characterisation of soluble and insoluble polymer fractions. Carbohydr. Polym..

[47] Peng X.-M., Huang L.-J., Qi C.-H., Zhang Y.-X., Tian G.-Y. (2010). Studies on chemistry and immuno-modulating mechanism of a glycoconjugate from *Lycium barbarum* L.. Chin. J. Chem..

[48] Peng X., Tian G. (2001). Structural characterization of the glycan part of glycoconjugate LbGp2 from *Lycium barbarum* L.. Carbohydr. Res..

[49] Huang L., Tian G., Qi C., Zhang Y. (2001). Structure elucidation and immunoactivity studies of glycan of glycoconjugate LbGp4 isolated from the fruit of *Lycium barbarum* L.. Chem. J. Chin. Univ..

[50] Chunhui Q., Linjuan H., Yongxiang Z., Xiunan Z., Gengyuan T., Xiangbin R. (2001). Chemical structure and immunoactivity of the glycoconjugates and their glycan chains from the fruit of *Lycium barbarum* L.. Chin. J. Pharmacol. Toxicol..

[51] Huang L.J., Tian G.Y., Ji G.Z. (1999). Elucidation of glycan of glycoconjugate LbGp3 isolated from the fruit of *Lycium barbarum* L.. J. Asian Nat. Prod. Res..

[52] Zhao C., Li R., He Y., Chui G. (1997). Studies on the chemistry of Gouqi polysaccharides. J. Beijing Med. Univ..

[53] Duan C.L., Qiao S.Y., Wang N.L., Zhao Y.M., Yao X.S. (2001). Studies on the active polysaccharides from *Lycium barbarum* L.. Yao Xue Xue Bao.

[54] Gan L., Zhang S.H., Liu Q., Xu H.B. (2003). A polysaccharide-protein complex from *Lycium barbarum* upregulates cytokine expression in human peripheral blood mononuclear cells. Eur. J. Pharmacol..

[55] Zhao C., He Y., Li R., Cui G. (1996). Chemistry and pharmacological activity of peptidoglycan from *Lycium barbarum* L.. Chin. Chem. Lett..

[56] Liu H., Fan Y., Wang W., Liu N., Zhang H., Zhu Z., Liu A. (2012). Polysaccharides from *Lycium barbarum* leaves: Isolation, characterization and splenocyte proliferation activity. Int. J. Biol. Macromol..

[57] Anderson C.T., Cohen E., Merzendorfer H. (2019). Pectic polysaccharides in plants: Structure, biosynthesis, functions, and applications. Extracellular Sugar-Based Biopolymers Matrices.

[58] Luis A.S., Briggs J., Zhang X., Farnell B., Ndeh D., Labourel A., Baslé A., Cartmell A., Terrapon N., Stott K. (2018). Dietary pectic glycans are degraded by coordinated enzyme pathways in human colonic *Bacteroides*. Nat. Microbiol..

[59] Ndeh D., Rogowski A., Cartmell A., Luis A.S., Baslé A., Gray J., Venditto I., Briggs J., Zhang X., Labourel A. (2017). Complex pectin metabolism by gut bacteria reveals novel catalytic functions. Nature.

[60] Colosimo R., Mulet-Cabero A.-I., Cross K.L., Haider K., Edwards C.H., Warren F.J., Finnigan T.J.A., Wilde P.J. (2021). β-glucan release from fungal and plant cell walls after simulated gastrointestinal digestion. J. Funct. Food..

[61] Prade R.A. (1996). Xylanases: From biology to biotechnology. Biotechnol. Genet. Eng. Rev..

[62] Wang Y., Sun M., Jin H., Yang J., Kang S., Liu Y., Yang S., Ma S., Ni J. (2021). Effects of *Lycium barbarum* polysaccharides on immunity and the gut microbiota in cyclophosphamide-induced immunosuppressed mice. Front. Microbiol..

[63] Eckburg P.B., Bik E.M., Bernstein C.N., Purdom E., Dethlefsen L., Sargent M., Gill S.R., Nelson K.E., Relman D.A. (2005). Diversity of the human intestinal microbial flora. Science.

[64] Louis P. (2017). Different substrate preferences help closely related bacteria to coexist in the gut. mBio.

[65] Rakoff-Nahoum S., Foster K.R., Comstock L.E. (2016). The evolution of cooperation within the gut microbiota. Nature.

[66] Garrett W.S., Gordon J.I., Glimcher L.H. (2010). Homeostasis and inflammation in the intestine. Cell.

[67] Koboziev I., Reinoso Webb C., Furr K.L., Grisham M.B. (2014). Role of the enteric microbiota in intestinal homeostasis and inflammation. Free Radic. Biol. Med..

[68] Shin N.R., Whon T.W., Bae J.W. (2015). Proteobacteria: Microbial signature of dysbiosis in gut microbiota. Trends Biotechnol..

[69] Wexler A.G., Goodman A.L. (2017). An insider’s perspective: *Bacteroides* as a window into the microbiome. Nat. Microbiol..

[70] Sartor R.B.M., Sarkis K. (2012). Intestinal microbes in inflammatory bowel diseases. Am. J. Gastroenterol. Suppl..

[71] Yu L.I., Zhou Y., Jiang G.M., Ding Y.X., Fang-Fei X.U., Wang Q. (2012). Study on the antibacterial activity of *Lycium barbarum* polysaccharide and *Astragalus* polysaccharide. Prog. Mod. Biomed..

[72] Chunyan G., Lizhu J., Chengrui T. (2007). Study on the antibacterial activity of Ch. Wolfberry polysaccharide. Food Sci. Technol..

[73] Wang J., Hu Y., Wang D., Zhang F., Zhao X., Abula S., Fan Y., Guo L. (2010). *Lycium barbarum* polysaccharide inhibits the infectivity of Newcastle disease virus to chicken embryo fibroblast. Int. J. Biol. Macromol..

[74] Deng X., Lin Q., Luo X., Zhou L. (2019). Effects of *Lycium barbarum* polysaccharide on intestinal *E. coli*, *Bifidobacteria* and *Lactobacillus* in H22 hepatocellular carcinoma mice. Food Sci. Technol..

[75] Hessle C.C., Andersson B., Wold A.E. (2005). Gram-positive and Gram-negative bacteria elicit different patterns of pro-inflammatory cytokines in human monocytes. Cytokine.

[76] Swanson L., Katkar G.D., Tam J., Pranadinata R.F., Chareddy Y., Coates J., Anandachar M.S., Castillo V., Olson J., Nizet V. (2020). TLR4 signaling and macrophage inflammatory responses are dampened by GIV/Girdin. Proc. Natl. Acad. Sci. USA.

[77] Sampath V. (2018). Bacterial endotoxin-lipopolysaccharide; structure, function and its role in immunity in vertebrates and invertebrates. Agric. Nat. Resour..

[78] Cao C., Zhu B., Liu Z., Wang X., Ai C., Gong G., Hu M., Huang L., Song S. (2021). An arabinogalactan from *Lycium barbarum* attenuates DSS-induced chronic colitis in C57BL/6J mice associated with the modulation of intestinal barrier function and gut microbiota. Food Funct..

[79] Gao L., Ma J., Fan Y., Zhang Y., Ge R., Tao X., Zhang M., Gao Q., Yang J. (2021). *Lycium barbarum* polysaccharide combined with aerobic exercise ameliorated nonalcoholic fatty liver disease through restoring gut microbiota, intestinal barrier and inhibiting hepatic inflammation. Int. J. Biol. Macromol..

[80] Van Zyl W.F., Deane S.M., Dicks L.M.T. (2020). Molecular insights into probiotic mechanisms of action employed against intestinal pathogenic bacteria. Gut Microbes.

[81] Zhu W., Zhou S., Liu J., McLean R., Chu W. (2019). Prebiotic, immuno-stimulating and gut microbiota-modulating effects of *Lycium barbarum* polysaccharide. Biomed. Pharmacother..

[82] Arzamasov A.A., van Sinderen D., Rodionov D.A. (2018). Comparative genomics reveals the regulatory complexity of Bifidobacterial arabinose and arabino-oligosaccharide utilization. Front. Microbiol..

[83] Thongaram T., Hoeflinger J.L., Chow J., Miller M.J. (2017). Prebiotic galactooligosaccharide metabolism by probiotic Lactobacilli and Bifidobacteria. J. Agric. Food Chem..

[84] Gullón B., Gómez B., Martínez-Sabajanes M., Yáñez R., Parajó J.C., Alonso J.L. (2013). Pectic oligosaccharides: Manufacture and functional properties. Trends Food Sci. Technol..

[85] Islam S.U. (2016). Clinical Uses of Probiotics. Medicine.

[86] Davani-Davari D., Negahdaripour M., Karimzadeh I., Seifan M., Mohkam M., Masoumi S.J., Berenjian A., Ghasemi Y. (2019). Prebiotics: Definition, types, sources, mechanisms, and clinical applications. Foods.

[87] Yu C., Hu X., Ahmadi S., Wu D., Xiao H., Zhang H., Ding T., Liu D., Ye X., Chen S. (2022). Structure and in vitro fermentation characteristics of polysaccharides sequentially extracted from Goji Berry (*Lycium barbarum*) leaves. J. Agric. Food Chem..

[88] Xia W., Li X., Khan I., Yin L., Su L., Leong W., Bian X., Su J.-Y., Hsiao W.L.W., Huang G. (2020). *Lycium* berry polysaccharides strengthen gut microenvironment and modulate gut microbiota of the mice. Evid.-Based Complement. Altern. Med..

[89] Sinha S.R., Haileselassie Y., Nguyen L.P., Tropini C., Wang M., Becker L.S., Sim D., Jarr K., Spear E.T., Singh G. (2020). Dysbiosis-induced secondary bile acid deficiency promotes intestinal inflammation. Cell Host Microbe.

[90] Tian B., Zhang Z., Zhao J., Ma Q., Liu H., Nie C., Ma Z., An W., Li J. (2021). Dietary whole Goji berry (*Lycium barbarum)* intake improves colonic barrier function by altering gut microbiota composition in mice. Int. J. Food Sci. Technol..

[91] Ding Y., Yan Y., Chen D., Ran L., Mi J., Lu L., Jing B., Li X., Zeng X., Cao Y. (2019). Modulating effects of polysaccharides from the fruits of *Lycium barbarum* on the immune response and gut microbiota in cyclophosphamide-treated mice. Food Funct..

[92] Zhao Y., Yan Y., Zhou W., Chen D., Huang K., Yu S., Mi J., Lu L., Zeng X., Cao Y. (2020). Effects of polysaccharides from bee collected pollen of Chinese wolfberry on immune response and gut microbiota composition in cyclophosphamide-treated mice. J. Funct. Food..

[93] Kayama H., Okumura R., Takeda K. (2020). Interaction between the microbiota, epithelia, and immune cells in the intestine. Annu. Rev. Immunol..

[94] Gasaly N., de Vos P., Hermoso M.A. (2021). Impact of bacterial metabolites on gut barrier function and host immunity: A focus on bacterial metabolism and its relevance for intestinal inflammation. Front. Immunol..

[95] McNabney S.M., Henagan T.M. (2017). Short chain fatty acids in the colon and peripheral tissues: A focus on butyrate, colon cancer, obesity and insulin resistance. Nutrients.

[96] Tazoe H., Otomo Y., Kaji I., Tanaka R., Karaki S.I., Kuwahara A. (2008). Roles of short-chain fatty acids receptors, GPR41 and GPR43 on colonic functions. J. Physiol. Pharmacol..

[97] Parada Venegas D., De la Fuente M.K., Landskron G., González M.J., Quera R., Dijkstra G., Harmsen H.J.M., Faber K.N., Hermoso M.A. (2019). Short chain fatty acids (SCFAs)-mediated gut epithelial and immune regulation and its relevance for inflammatory bowel diseases. Front. Immunol..

[98] Jin U., Cheng Y., Park H., Davidson L.A., Callaway E.S., Chapkin R.S., Jayaraman A., Asante A., Allred C., Weaver E.A. (2017). Short chain fatty acids enhance aryl hydrocarbon (Ah) responsiveness in mouse colonocytes and Caco-2 human colon cancer cells. Sci. Rep..

[99] Sun M., Wu W., Liu Z., Cong Y. (2017). Microbiota metabolite short chain fatty acids, GPCR, and inflammatory bowel diseases. J. Gastroenterol..

[100] Chen J., Long L., Jiang Q., Kang B., Li Y., Yin J. (2020). Effects of dietary supplementation of *Lycium barbarum* polysaccharides on growth performance, immune status, antioxidant capacity and selected microbial populations of weaned piglets. J. Anim. Physiol. Anim. Nutr..

[101] Lu H., Liu P., Zhang X., Bao T., Wang T., Guo L., Li Y., Dong X., Li X., Dong Y. (2021). Inulin and *Lycium barbarum* polysaccharides ameliorate diabetes by enhancing gut barrier via modulating gut microbiota and activating gut mucosal TLR2+ intraepithelial γδ T cells in rats. J. Funct. Food..

[102] Cui F., Shi C.L., Zhou X.J., Wen W., Gao X.P., Wang L.Y., He B., Yin M., Zhao J.Q. (2020). *Lycium barbarum* polysaccharide extracted from *Lycium barbarum* leaves ameliorates asthma in mice by reducing inflammation and modulating gut microbiota. J. Med. Food.

[103] Yang M., Yin Y., Wang F., Zhang H., Ma X., Yin Y., Tan B., Chen J. (2021). Supplementation with *Lycium barbarum* polysaccharides reduce obesity in high-fat diet-fed mice by modulation of gut microbiota. Front. Microbiol..

[104] Cao C., Wang L., Ai C., Gong G., Wang Z., Huang L., Song S., Zhu B. (2022). Impact of *Lycium barbarum* arabinogalactan on the fecal metabolome in a DSS-induced chronic colitis mouse model. Food Funct..

[105] Zhou W., Yang T., Xu W., Huang Y., Ran L., Yan Y., Mi J., Lu L., Sun Y., Zeng X. (2022). The polysaccharides from the fruits of *Lycium barbarum* L. confer anti-diabetic effect by regulating gut microbiota and intestinal barrier. Carbohydr. Polym..

[106] Xia H., Tang H., Wang F., Yang X., Wang Z., Liu H., Pan D., Yang C., Wang S., Sun G. (2019). An untargeted metabolomics approach reveals further insights of *Lycium barbarum* polysaccharides in high fat diet and streptozotocin-induced diabetic rats. Food Res. Int..

[107] Wang H., Zhang S., Shen Q., Zhu M.J. (2019). A metabolomic explanation on beneficial effects of dietary Goji on intestine inflammation. J. Funct. Food..

[108] Ding Y., Chen D., Yan Y., Chen G., Ran L., Mi J., Lu L., Zeng X., Cao Y. (2021). Effects of long-term consumption of polysaccharides from the fruit of *Lycium barbarum* on host’s health. Food Res. Int..

[109] Liu Y., Fang H., Liu H., Cheng H., Pan L., Hu M., Li X. (2021). Goji berry juice fermented by probiotics attenuates dextran sodium sulfate-induced ulcerative colitis in mice. J. Funct. Food..

[110] Zhao X.Q., Guo S., Lu Y.Y., Hua Y., Zhang F., Yan H., Shang E.X., Wang H.Q., Zhang W.H., Duan J.A. (2020). *Lycium barbarum* L. leaves ameliorate type 2 diabetes in rats by modulating metabolic profiles and gut microbiota composition. Biomed. Pharmacother..

[111] Liu H., Zhang Z., Li J., Liu W., Warda M., Cui B., Abd El-Aty A.M. (2022). Oligosaccharides derived from *Lycium barbarum* ameliorate glycolipid metabolism and modulate the gut microbiota community and the faecal metabolites in a type 2 diabetes mouse model: Metabolomic bioinformatic analysis. Food Funct..

[112] Fan Y., Yan L., Li M., Pu Z., Zhang Y., Yang J. (2021). *Lycium barbarum* polysaccharides regulate the gut microbiota to modulate metabolites in high fat diet-induced obese rats. Res. Sq..

[113] Zhao F., Guan S., Fu Y., Wang K., Liu Z., Ng T.B. (2021). *Lycium barbarum* polysaccharide attenuates emotional injury of offspring elicited by prenatal chronic stress in rats via regulation of gut microbiota. Biomed. Pharmacother..

[114] Zheng Y., Pang X., Zhu X., Meng Z., Chen X., Zhang J., Ding Q., Li Q., Dou G., Ma B. (2021). *Lycium barbarum* mitigates radiation injury via regulation of the immune function, gut microbiota, and related metabolites. Biomed. Pharmacother..

[115] Zhang Z., Liu H., Yu B., Tao H., Li J., Wu Z., Liu G., Yuan C., Guo L., Cui B. (2020). *Lycium barbarum* polysaccharide attenuates myocardial injury in high-fat diet-fed mice through manipulating the gut microbiome and fecal metabolome. Food Res. Int..

[116] Lian Y.Z., Lin I.H., Yang Y.-C., Chao J.C.J. (2020). Gastroprotective effect of *Lycium barbarum* polysaccharides and C-phycocyanin in rats with ethanol-induced gastric ulcer. Int. J. Biol. Macromol..

[117] Skenderidis P., Mitsagga C., Lampakis D., Petrotos K., Giavasis I. (2019). The effect of encapsulated powder of Goji berry (*Lycium barbarum*) on growth and survival of probiotic bacteria. Microorganisms.

[118] Zhou W., Yan Y., Mi J., Zhang H., Lu L., Luo Q., Li X., Zeng X., Cao Y. (2018). Simulated digestion and fermentation in vitro by human gut microbiota of polysaccharides from bee collected pollen of Chinese Wolfberry. J. Agric. Food Chem..

[119] Rakoff-Nahoum S., Coyne M.J., Comstock L.E. (2014). An ecological network of polysaccharide utilization among human intestinal symbionts. Curr. Biol..

[120] Bhatia S., Prabhu P.N., Benefiel A.C., Miller M.J., Chow J., Davis S.R., Gaskins H.R. (2015). Galacto-oligosaccharides may directly enhance intestinal barrier function through the modulation of goblet cells. Mol. Nutr. Food Res..

[121] Figueroa-Lozano S., Ren C., Yin H., Pham H., van Leeuwen S., Dijkhuizen L., de Vos P. (2020). The impact of oligosaccharide content, glycosidic linkages and lactose content of galacto-oligosaccharides (GOS) on the expression of mucus-related genes in goblet cells. Food Funct..

[122] Difilippo E., Bettonvil M., Willems R., Braber S., Fink-Gremmels J., Jeurink P.V., Schoterman M.H.C., Gruppen H., Schols H.A. (2015). Oligosaccharides in urine, blood, and feces of piglets fed milk replacer containing galacto-oligosaccharides. J. Agric. Food Chem..

[123] Vazquez E., Santos-Fandila A., Buck R., Rueda R., Ramirez M. (2017). Major human milk oligosaccharides are absorbed into the systemic circulation after oral administration in rats. Br. J. Nutr..

[124] Bäckhed F., Ley R.E., Sonnenburg J.L., Peterson D.A., Gordon J.I. (2005). Host-bacterial mutualism in the human intestine. Science.

[125] Nicholson J.K., Holmes E., Kinross J., Burcelin R., Gibson G., Jia W., Pettersson S. (2012). Host-gut microbiota metabolic interactions. Science.

[126] Xiao Z., Deng Q., Zhou W., Zhang Y. (2022). Immune activities of polysaccharides isolated from *Lycium barbarum* L. What do we know so far?. Pharmacol. Ther..

[127] Bo R., Liu Z., Zhang J., Gu P., Ou N., Sun Y., Hu Y., Liu J., Wang D. (2019). Mechanism of *Lycium barbarum* polysaccharides liposomes on activating murine dendritic cells. Carbohydr. Polym..

[128] McGuckin M., Eri R., Simms L.A., Md T., Dphil G. (2009). Intestinal barrier dysfunction in inflammatory bowel diseases. Inflamm. Bowel Dis..

[129] Hayashi T., Ishida T., Motoya S., Itoh F., Takahashi T., Hinoda Y., Imai K. (2001). Mucins and immune reactions to mucins in ulcerative colitis. Digestion.

[130] Strous G.J., Dekker J. (1992). Mucin-type glycoproteins. Crit. Rev. Biochem. Mol. Biol..

[131] Qu D., Wang G., Yu L., Tian F., Chen W., Zhai Q. (2021). The effects of diet and gut microbiota on the regulation of intestinal mucin glycosylation. Carbohydr. Polym..

[132] Bergstrom K., Fu J., Johansson M.E.V., Liu X., Gao N., Wu Q., Song J., McDaniel J.M., McGee S., Chen W. (2017). Core 1- and 3- derived *O*-glycans collectively maintain the colonic mucus barrier and protect against spontaneous colitis in mice. Mucosal Immunol..

[133] Paone P., Cani P.D. (2020). Mucus barrier, mucins and gut microbiota: The expected slimy partners?. Gut.

[134] Desai M., Seekatz A., Koropatkin N., Kamada N., Martens E. (2016). A dietary fiber-deprived gut microbiota degrades the colonic mucus barrier and enhances pathogen susceptibility. Cell.

[135] Vivinus-Nébot M., Frin-Mathy G., Bzioueche H., Dainese R., Bernard G., Anty R., Filippi J., Saint-Paul M.C., Tulic M.K., Verhasselt V. (2014). Functional bowel symptoms in quiescent inflammatory bowel diseases: Role of epithelial barrier disruption and low-grade inflammation. Gut.

[136] Schulzke J.D., Ploeger S., Amasheh M., Fromm A., Zeissig S., Troeger H., Richter J., Bojarski C., Schumann M., Fromm M. (2009). Epithelial tight junctions in intestinal inflammation. Ann. N. Y. Acad. Sci..

[137] Van der Sluis M., De Koning B.A., De Bruijn A.C., Velcich A., Meijerink J.P., Van Goudoever J.B., Büller H.A., Dekker J., Van Seuningen I., Renes I.B. (2006). MUC2-deficient mice spontaneously develop colitis, indicating that MUC2 is critical for colonic protection. Gastroenterology.

[138] Capaldo C.T., Powell D.N., Kalman D. (2017). Layered defense: How mucus and tight junctions seal the intestinal barrier. J. Mol. Med..

[139] Pan L., Fu T., Cheng H., Mi J., Shang Q., Yu G. (2022). Polysaccharide from edible alga *Gloiopeltis furcata* attenuates intestinal mucosal damage by therapeutically remodeling the interactions between gut microbiota and mucin O-glycans. Carbohydr. Polym..

[140] Round J.L., Mazmanian S.K. (2009). The gut microbiota shapes intestinal immune responses during health and disease. Nat. Rev. Immunol..

[141] Chervonsky A.V. (2012). Intestinal commensals: Influence on immune system and tolerance to pathogens. Curr. Opin. Immunol..

[142] Walter J., Armet A.M., Finlay B.B., Shanahan F. (2020). Establishing or exaggerating causality for the gut microbiome: Lessons from human microbiota-associated rodents. Cell.

[143] Xiao T.S. (2017). Innate immunity and inflammation. Cell. Mol. Immunol..

[144] Barthels C., Ogrinc A., Steyer V., Meier S., Simon F., Wimmer M., Blutke A., Straub T., Zimber-Strobl U., Lutgens E. (2017). CD40-signalling abrogates induction of RORγt^+^ Treg cells by intestinal CD103^+^ DCs and causes fatal colitis. Nat. Commun..

[145] Li Z., Lin L., Hao Q., Zhai H. (2022). Effects of *Lycium barbarum* polysaccharide on intestinal microbiota regulation in ulcerative colitis mice. J. Nutr. Metab. Cancer.

[146] Serino M., Luche E., Gres S., Baylac A., Bergé M., Cenac C., Waget A., Klopp P., Iacovoni J., Klopp C. (2012). Metabolic adaptation to a high-fat diet is associated with a change in the gut microbiota. Gut.

[147] Zmora N., Bashiardes S., Levy M., Elinav E. (2017). The role of the immune system in metabolic health and disease. Cell Metab..

[148] Magne F., Gotteland M., Gauthier L., Zazueta Hernández A., Pesoa S., Navarrete P., Balamurugan R. (2020). The Firmicutes/Bacteroidetes ratio: A relevant marker of gut dysbiosis in obese patients?. Nutrients.

[149] Li Q., Hagberg C.E., Silva Cascales H., Lang S., Hyvönen M.T., Salehzadeh F., Chen P., Alexandersson I., Terezaki E., Harms M.J. (2021). Obesity and hyperinsulinemia drive adipocytes to activate a cell cycle program and senesce. Nat. Med..

[150] Everard A., Belzer C., Geurts L., Ouwerkerk J.P., Druart C., Bindels L.B., Guiot Y., Derrien M., Muccioli G.G., Delzenne N.M. (2013). Cross-talk between *Akkermansia muciniphila* and intestinal epithelium controls diet-induced obesity. Proc. Natl. Acad. Sci. USA.

[151] Mitra S., De A., Chowdhury A. (2020). Epidemiology of non-alcoholic and alcoholic fatty liver diseases. Transl. Gastroenterol. Hepatol..

[152] Brunt E.M., Wong V.W.S., Nobili V., Day C.P., Sookoian S., Maher J.J., Bugianesi E., Sirlin C.B., Neuschwander-Tetri B.A., Rinella M.E. (2015). Nonalcoholic fatty liver disease. Nat. Rev. Dis. Primers.

[153] Yang X., Bai H., Cai W., Li J., Zhou Q., Wang Y., Han J., Zhu X., Dong M., Hu D. (2013). *Lycium barbarum* polysaccharides reduce intestinal ischemia/reperfusion injuries in rats. Chem. Biol. Interact..

[154] Xue L., He J., Gao N., Lu X., Li M., Wu X., Liu Z., Jin Y., Liu J., Xu J. (2017). Probiotics may delay the progression of nonalcoholic fatty liver disease by restoring the gut microbiota structure and improving intestinal endotoxemia. Sci. Rep..

[155] Lu Y., Fan C., Li P., Lu Y., Chang X., Qi K. (2016). Short chain fatty acids prevent high-fat-diet-induced obesity in mice by regulating G protein-coupled receptors and gut microbiota. Sci. Rep..

[156] Tripathi A., Debelius J., Brenner D.A., Karin M., Loomba R., Schnabl B., Knight R. (2018). The gut-liver axis and the intersection with the microbiome. Nat. Rev. Gastroenterol. Hepatol..

[157] Chen L., Li W., Qi D., Wang D. (2018). *Lycium barbarum* polysaccharide protects against LPS-induced ARDS by inhibiting apoptosis, oxidative stress, and inflammation in pulmonary endothelial cells. Free Radic. Res..

[158] Morais L.H., Schreiber H.L., Mazmanian S.K. (2021). The gut microbiota-brain axis in behaviour and brain disorders. Nat. Rev. Microbiol..

[159] Li X., Mo X., Liu T., Shao R., Teopiz K., McIntyre R.S., So K.F., Lin K. (2022). Efficacy of *Lycium barbarum* polysaccharide in adolescents with subthreshold depression: Interim analysis of a randomized controlled study. Neural Regen Res..

